# Serum dysregulation of serine and glycine metabolism as predictive biomarker for cognitive decline in frail elderly subjects

**DOI:** 10.1038/s41398-024-02991-z

**Published:** 2024-07-09

**Authors:** Alberto Imarisio, Isar Yahyavi, Clara Gasparri, Amber Hassan, Micol Avenali, Anna Di Maio, Gabriele Buongarzone, Caterina Galandra, Marta Picascia, Asia Filosa, Maria Cristina Monti, Claudio Pacchetti, Francesco Errico, Mariangela Rondanelli, Alessandro Usiello, Enza Maria Valente

**Affiliations:** 1 Department of Molecular Medicine, University of Pavia, Pavia, Italy; 2 Neurogenetics Research Centre, IRCCS Mondino Foundation, Pavia, Italy; 3 Department of Environmental, Biological and Pharmaceutical Sciences and Technologies, Università degli Studi della Campania “Luigi Vanvitelli”, Caserta, Italy; 4 CEINGE Biotecnologie Avanzate Franco Salvatore, Naples, Italy; 5 Endocrinology and Nutrition Unit, Azienda di Servizi alla Persona “Istituto Santa Margherita”, University of Pavia, Pavia, Italy; 6 Department of Brain and Behavioral Sciences, University of Pavia, Pavia, Italy; 7 Parkinson’s Disease and Movement Disorders Unit, IRCCS Mondino Foundation, Pavia, Italy; 8 Department of Public Health, Experimental and Forensic Medicine, University of Pavia, Pavia, Italy; 9 Department of Agricultural Sciences, University of Naples “Federico II”, Portici, Italy

**Keywords:** Diagnostic markers, Molecular neuroscience, Long-term memory, Depression, Prognostic markers

## Abstract

Frailty is a common age-related clinical syndrome characterized by a decline in the function of multiple organ systems, increased vulnerability to stressors, and a huge socio-economic burden. Despite recent research efforts, the physiopathological mechanisms underlying frailty remain elusive and biomarkers able to predate its occurrence in the early stages are still lacking. Beyond its physical component, cognitive decline represents a critical domain of frailty associated with higher risk of adverse health outcomes. We measured by High-Performance Liquid Chromatography (HPLC) a pool of serum amino acids including L-glutamate, L-aspartate, glycine, and D-serine, as well as their precursors L-glutamine, L-asparagine, and L-serine in a cohort of elderly subjects encompassing the entire continuum from fitness to frailty. These amino acids are known to orchestrate excitatory and inhibitory neurotransmission, and in turn, to play a key role as intermediates of energy homeostasis and in liver, kidney, muscle, and immune system metabolism. To comprehensively assess frailty, we employed both the Edmonton Frail Scale (EFS), as a practical tool to capture the multidimensionality of frailty, and the frailty phenotype, as a measure of physical function. We found that D-serine and D-/Total serine ratio were independent predictors of EFS but not of physical frailty. Furthermore, higher levels of glycine, glycine/L-serine and D-/Total serine were associated with worse cognition and depressive symptoms in the frail group. These findings suggest that changes in peripheral glycine and serine enantiomers homeostasis may represent a novel biochemical correlate of frailty.

## Introduction

Frailty is a complex clinical syndrome characterized by a progressive deterioration of physiological function of multiple organ systems, with consequent increased vulnerability to stressors and adverse health outcomes [1]. Frailty is common in elderly populations, with a prevalence in high-income countries ranging from 4 to 16 percent in people over 65 years of age and featuring a two-fold higher risk in women than in men [2–4].

Frailty is recognized as a main determinant of disability, institutionalization, and mortality among older people. However, frailty also represents a dynamic condition which exists on a continuum from fit to frail, where a subject’s status can change in either direction over time [1]. Previous longitudinal studies showed indeed that up to 57% of individuals experience at least one transition, which includes both worsening, and improvement in frailty state [4, 5]. This evidence suggests that the factors concurring to determine frailty may be targeted with preventive interventions to reduce its burden on health outcomes.

Heterogeneous frailty definitions and operational scales have been proposed, with large variations in their biological rationale and included components [6]. Among the most commonly adopted, Fried’s frailty phenotype considers frailty as a biological syndrome and classifies individuals on the basis of five physical components [7]. A few years later, Rolfson and colleagues proposed the Edmonton Frail Scale (EFS), a brief and point-of-care frailty evaluation tool whose reliability is comparable to the most comprehensive geriatric assessment scales [8, 9]. Among its nine items, the EFS includes an assessment of primary brain-related functions including cognition, mood, and social support, whose impairment represents a key component of frailty and is associated with increased social isolation, disability, and mortality [10, 11].

Despite the physiopathological mechanisms responsible for frailty still remain elusive, frailty prevalence, and incidence have been linked to several defective physiological processes, including alterations in insulin resistance, energy-regulatory hormones, musculoskeletal system function, and mitochondrial energy production, autonomic nervous system and systemic inflammation [12]. Consistent with the complex nature of frailty syndrome, several alterations involving multiple pathways and cellular processes in distinct organs have been disclosed by OMICS approaches [13]. In particular, recent metabolomics studies described biochemical alterations in frail subjects, including variations in anti-oxidant, inflammation, purine, urea cycle, kidney markers, tricarboxylic acid cycle, and amino acid pathways [14–24]. The discovery of reliable biomarkers of frailty represents, therefore, a key milestone for identifying and monitoring the course of this syndrome along aging and, in turn, offering a possible therapeutic approach aimed at reverting frailty. However, previous OMICS results are inconsistent among independent studies [13] and, except for pro-inflammatory soluble cytokines, which are commonly increased in older frail subjects [25], a unified biochemical marker representative of this syndrome is currently lacking. Moreover, given the critical relevance of cognitive decline and mood alterations reported in frailty [11, 26, 27], the identification of a specific biochemical hallmark mirroring the progressive decay of brain functions before the occurrence of overt dementia represents an unmet clinical need.

Previous blood metabolomics studies identified a dysregulation in the homeostasis of glutamate pathway in frail individuals compared to controls [14–18, 21]. Moreover, altered glutamate and aspartate metabolism has been associated in independent cohorts to sarcopenia [28–30], which represents one of the core features of the physical domain of frailty. In light of these findings, here we measured by High-Performance Liquid Chromatography (HPLC) a pool of amino acids that collectively are known to modulate glutamatergic receptors (GluR) activation (L-glutamate, L-aspartate, glycine, D-serine) or to represent the immediate precursors of these neuroactive molecules (L-glutamine, L-asparagine, and L-serine) in a well-characterized cohort of elderly subjects encompassing the entire continuum from non-frail to frail condition. Noteworthy, in addition to their neuroactive role, these amino acids play critical roles in regulating various cellular pathways, including protein synthesis, tricarboxylic acid cycle, redox homeostasis, ammonium recycling, purine nucleotide cycle, folate and methionine cycles, and the synthesis of sphingolipids and phospholipids [31]. Consistently, these biomolecules have a vital relevance in orchestrating cognition, mood, energy homeostasis, and immune system functions, as well as the metabolism of various peripheral organs, such as skeletal muscles, liver, and kidney [12, 26, 32, 33]. Given that the homeostasis of these systems and organs is severely affected in frail subjects, we investigated the relationship between the serum levels of these amino acids and frailty. We assessed frailty status with (i) the EFS score, which we adopted as a reliable instrument mirroring the multidimensionality of frailty [8]; (ii) the Fried’s phenotype, as a well-established tool to evaluate the physical domain of frailty [7]. We also took into account the effect of several comorbidities and health parameters representing key components of frailty and potentially impacting the blood levels of amino acids, including body mass index (BMI), visceral adipose tissue (VAT), sarcopenia, diabetes mellitus, and cigarette smoking.

## Methods

### Participants

#### Enrollment and inclusion/exclusion criteria

Forty-five consecutive hospitalized subjects were recruited at the Physical Medicine and Rehabilitation Unit of Istituto Santa Margherita, Pavia, Italy, between February 2019 and August 2021. Eighty additional outpatients were recruited at the Endocrinology and Nutrition Unit of the same institute. The patients were included if (1) admitted for functional loss secondary to a non-disabling disease; (2) aged 65 years or older. The following exclusion criteria were applied: (1) any disease that could directly affect muscle strength (including neurological diseases, hip fractures or amputations); (2) dementia according to DSM-5 criteria [34]; (3) any systemic condition potentially affecting serum amino acid levels, including kidney, liver, rheumatologic and neoplastic diseases, history of drug or alcohol abuse; (4) history of altered serum creatinine levels (>1.2 mg/dl) or liver function parameters (aspartate transaminase or alanine transaminase >50 U/l).

Smoking status (current/former/never smoker) was assessed trough interview. The total number of drugs habitually taken by subjects was retrieved from medical records. This study was approved by the local ethics committee (protocol 20180097520, 09/11/2018) and was in conformity with the Helsinki Declaration. Written informed consent was obtained from all participants.

#### Cognitive and mood evaluation

Each subject underwent a standardized examination including evaluation of global cognition, performed through the Mini-Mental State Examination (MMSE) and Montreal Cognitive Assessment (MoCA) [35], and of depressive symptoms, measured with the Hamilton Depression Rating Scale (HAM-D) [36].

#### Quality of life

Quality of life was assessed through the Italian validation of the 36-Item Short Form Survey (SF-36) [37]. The arithmetic mean of the scores obtained in the nine scales of SF-36 was used as a global measure to compare the quality of life between non-frail and frail groups. We used the General Health scale score of SF-36 as a single frailty domain to be correlated with serum amino acids levels, since it is the SF-36 scale semantically closer to the General Health Status item of the EFS [8].

#### Sarcopenia and visceral adiposity

Body composition (fat mass (FM) and fat-free mass (FFM)) was evaluated using fan‐beam dual‐energy X‐ray absorptiometry (DXA) (Lunar Prodigy DXA, GE Medical Systems). The in vivo coefficients of variation were 0.89 and 0.48% for FM and FFM, respectively. Skeletal Muscle Index (SMI) was calculated as the sum of fat-free soft tissue mass of arms and legs divided for height squared [38]. Visceral adipose tissue (VAT) volume was estimated using a constant correction factor (0.94 g/cm^3^). The software automatically placed a quadrilateral box, representing the android region, outlined by the iliac crest and with a superior height equivalent to 20% of the distance from the top of the iliac crest to the base of the skull [39].

#### Functional performance and independence

Handgrip strength test was performed using a Jamar dynamometer adhering to the standardized protocol recommended by the American Society of Hand Therapists [40]. Handgrip measurement was assessed on the dominant hand and was considered “strong” or “weak” based on sex and body mass index (BMI)-adjusted cut-off scores, as previously described [7]. Basic Activities of Daily Living (BADL) and Independent Activities of Daily Living (IADL) were measured by interviewing the patients and caregivers [41].

#### Nutritional status

Nutritional status was evaluated with Mini Nutritional Assessment (MNA), which is composed of 18 items divided in four categories: anthropometric assessment, general state, dietary assessment, and self-assessment. A score ≥ 24 points indicates a good nutritional status; a score between 17 and 23.5 points indicates risk of malnutrition, while a score ≤ 17 points indicates malnutrition [42].

#### Frailty

Frailty was separately evaluated with the EFS and the frailty phenotype. The EFS assesses nine frailty domains (cognition, general health, functional independence, social support, medication usage, nutrition, mood, continence, functional performance) [8]. EFS score ranges from 0 to 17. Participants were classified as “non-frail” (EFS ≤ 5) or “frail” (EFS > 5) according to previously proposed cut-off [9]. Since only three subjects had an EFS score >11 (used to define the “severe frail” category [9]), we considered all the subjects with an EFS score > 5 as a single “frail” group.

The physical frailty phenotype contains 5 criteria, including weight loss, exhaustion, low physical activity, slow walking speed, and low grip strength [7]. Participants who met 3 or more criteria were defined “frail”, those who met 1 or 2 criteria were classified as “pre-frail” and those who met no criteria were defined “non-frail”.

### Collection and storage of serum samples

Blood sampling was performed after a 12-h fasting. Whole blood was collected by peripheral venipuncture into clot activator tubes and gently mixed. Sample was stored upright for 30 min at room temperature to allow blood to clot, and centrifuged at 2000 × *g* for 10 min at room temperature. Serum was aliquoted (0.5 ml) in polypropylene cryotubes and stored at –80 °C before usage.

### HPLC analysis of amino acids content

Serum samples were analyzed as previously reported [43]. Briefly, serum samples (100 µl) were mixed in a 1:10 dilution with HPLC-grade methanol (900 µl) and centrifuged at 13,000 × *g* for 10 min; supernatants were dried and then suspended in 0.2 M trichloroacetic acid (TCA). TCA supernatants were then neutralized with 0.2 M NaOH and subjected to precolumn derivatization with o-phthaldialdehyde /N-acetyl-L-cysteine in 50% methanol. Amino acids derivatives were resolved on a UHPLC Nexera X3 system (Shimadzu) by using a Shim-pack GIST C18 3-μm reversed-phase column (Shimadzu, 4.0 × 150 mm) under isocratic conditions (0.1 M sodium acetate buffer, pH 6.2, 1% tetrahydrofuran, and 1 ml/min flow rate). A washing step in 0.1 M sodium acetate buffer, 3% tetrahydrofuran, and 47% acetonitrile, was performed after every run. Identification and quantification of amino acids were based on retention times and peak areas, compared with those associated with external standards. Each HPLC experiment was performed in duplicate. The detected amino acids concentration was expressed as µM. Researchers who performed the HPLC analyses were blinded to the clinical status of participants.

### Statistical analyses

Based on previous works assessing the serum levels of the same metabolites investigated in this study [44–46], we considered a sample size (n) equal to 125 (with at least n = 50 for both frail and non-frail groups) adapt to ensure adequate power and a medium effect size in between-group comparisons. Clinical and demographic characteristics were described using, as summary statistics, median and the interquartile range (IQR) or absolute and relative frequencies. The comparison of clinical-demographic features between non-frail and frail groups were performed with Mann-Whitney U test (for binary EFS-based stratification) or Kruskal-Wallis test (for the three frailty phenotype categories) for continuous variables and Chi-square test for categorical variables. The normality of data distribution was checked with the Kolmogorov–Smirnov test. Due to non-normal distribution, the serum amino acid levels were log_10_-transformed and then compared between frail and non-frail groups using a four-way ANCOVA model with “frailty status”, “sex”, “type 2 diabetes”, and “smoking” as factors and “age” and “BMI” as covariates. Levene’s test was used to check the equality of variances between groups.

The correlation of serum amino acid concentration with age was evaluated with Spearman’s correlation test. Partial correlation analyses adjusted for the effect of age and sex were adopted to test the correlation between serum amino acid levels and EFS score and the other clinical variables. To assess the ability of serum amino acids levels to predict EFS score, we used multiple linear regression models including age, sex, the clinical predictors of EFS score [9], and the single amino acid concentrations as predictors and EFS score as dependent variable. To evaluate the ability of serum amino acids to predict the physical frailty phenotype [7] we adopted multinomial logistic regression models using age, sex, and the single amino acid concentration as predictors and frailty category as dependent variable. For linear regression analyses, we verified that the residuals were normally distributed, there was no heteroscedasticity, and no multicollinearity between the variables (variance inflation factor < 5). The latter was also evaluated in the logistic regression analyses. Significance was set at p < 0.05 for all analyses. All the statistical tests were two-sided. Data were analyzed by using SPSS 26.0 software (IBM, Armonk, NY, USA).

## Results

### Participants

One-hundred and twenty-five consecutive elderly subjects were enrolled in the study. The participants were stratified into non-frail (n = 74) and frail (n = 51) groups accordingly to EFS score [10]. Demographic and clinical features of study participants are reported in Table 1A. Frail subjects were older and showed higher females prevalence than non-frail participants. As expected, frail group showed worse performance in physical, sarcopenia, cognitive, nutritional, functional independence, and quality of life domains. Total medication count was higher in frail compared to non-frail group. The proportions of patients with type 2 diabetes mellitus and of current/former/never smokers were similar between non-frail and frail subjects.

**Table 1:** (A) Clinical and demographic features of elderly cohort considered as a whole and after stratification by frailty status according to EFS. (B) Serum amino acid levels in elderly cohort considered as a whole and after stratification by frailty status.

A) Frail vs non-frail: clinical-demographic features					
	N	Total (N = 125)	Non-frail	Frail	p
Age, years	74 NF, 51 FR	74.0 (69.5–81.0)	72.0 (68.0–75.0)	81.0 (75.0–85.0)	**<0.001**^**a**^
Female sex, n (%)	74 NF, 51 FR	95 (76.0)	51 (68.9)	44 (86.3)	**0.026**^**b**^
SPPB total score	74 NF, 51 FR	8.0 (5.0–10.0)	9.0 (8.0–10.0)	4.0 (3.0–7.0)	**<0.001**^**a**^
Handgrip (kg)	74 NF, 51 FR	20.0 (16.0–26.0)	24.0 (20.0–32-0)	16.0 (12.0–20.0)	**<0.001**^**a**^
SMI (kg/m^2^)	74 NF, 51 FR	7.6 (7.1–8.6)	8.1 (7.1–8.9)	7.5 (6.9–8.1)	**0.012**^**a**^
MMSE	70 NF, 48 FR	27.1 (26.0–27.7)	27.2 (26.2–27.7)	27.1 (25.7–27.7)	0.374^a^
MoCA	70 NF, 48 FR	24.1 (21.5–26.1)	25.3 (23.4–26.7)	21.4 (19.7–25.1)	**<0.001**^**a**^
MNA	74 NF, 50 FR	23.8 (20.6–25.5)	25.0 (23.5–26.0)	20.5 (18.5–23.1)	**<0.001**^**a**^
BADL	72 NF, 46 FR	6.0 (6.0–6.0)	6.0 (6.0–6.0)	6.0 (5.0–6.0)	**0.001**^**a**^
IADL	72 NF, 46 FR	8.0 (6.0–8.0)	8.0 (8.0–8.0)	6.0 (4.0–8.0)	**<0.001**^**a**^
HAM-D	72 NF, 46 FR	5.0 (2.0–10.0)	5.0 (2.0–9.8)	4.0 (2.0–12.0)	0.797^a^
SF-36 (mean score)	71 NF, 46 FR	66.8 (52.7–78.3)	73.7 (57.4–81.6)	61.9 (38.1–67.4)	**<0.001**^**a**^
Number of drugs	72 NF, 49 FR	4.0 (2.5–8.0)	3.0 (2.0–5.0)	7.0 (5.0–11.5)	**<0.001**^**a**^
Type 2 diabetes, n (%)	74 NF, 51 FR	21 (16.8)	10 (13.5)	11 (21.6)	0.236^b^
BMI (kg/m^2^)	74 NF, 51 FR	27.7 (24.2–32.5)	27.9 (24.2–31.7)	27.6 (23.7–33.3)	0.752^a^
VAT (g)	73 NF, 51 FR	1035 (548–1557)	1049 (530–1652)	960 (555–1502)	0.463^a^
Current smokers, n (%)	74 NF, 51 FR	15 (12.0)	9 (12.2)	6 (11.8)	0.152^b^
Former smokers, n (%)	74 NF, 51 FR	22 (17.6)	9 (12.2)	13 (25.5)	
Never smokers, n (%)	74 NF, 51 FR	88 (70.4)	56 (75.7)	32 (62.7)	
EFS	74 NF, 51 FR	4.0 (2.0–7.0)	2.0 (1.0–4.0)	8.0 (6.0–9.0)	**<0.001**^**a**^

B) Frail vs non-frail: serum amino acid levels					
	N	Total (N = 125)	Non-frail	Frail	p^c^
L-aspartate (μM)	74 NF, 51 FR	4.0 (3.0–5.6)	3.9 (3.0–5.5)	4.4 (3.1–6.5)	0.409
L-asparagine (μM)	74 NF, 51 FR	24.1 (19.8–34.3)	24.8 (20.8–28.0)	22.6 (19.2–28.8)	0.676
Glycine (μM)	74 NF, 51 FR	208.9 (174.0–288.2)	201.0 (161.8–268.3)	222.3 (185.6–400.4)	0.223
D-serine (μM)	74 NF, 51 FR	1.9 (1.6–2.3)	1.8 (1.5–2.1)	2.1 (1.7–2.6)	0.167
L-serine (μM)	74 NF, 51 FR	72.5 (60.0–88.9)	75.8 (62.3–89.5)	71.1 (54.5–85.1)	0.963
Glycine/L-serine	74 NF, 51 FR	2.9 (2.2–4.1)	2.6 (2.1–3.4)	3.2 (2.4–4.9)	0.270
D-/Total serine (%)	74 NF, 51 FR	2.5 (2.0–3.2)	2.3 (1.9–2.8)	2.8 (2.2–4.0)	0.181
L-glutamate (μM)	74 NF, 51 FR	26.7 (19.1–34.3)	25.3 (18.8–33.7)	28.3 (19.2–34.6)	0.299
L-glutamine (μM)	74 NF, 51 FR	323.0 (280.5–370.3)	325.3 (282.0–365.7)	316.5 (276.3–381.5)	0.456
L-glutamine/L-glutamate	74 NF, 51 FR	12.1 (9.8–16.7)	12.6 (10.1–16.6)	11.2 (9.8–17.4)	0.578

Data are shown as median (IQR) or absolute frequency (%) for continuous and categorical variables, respectively. The total number of non-frail (NF) and frail (FR) subjects for which data were available is reported in the second column. Significant *p*-values are shown in bold.

*BADL* basic activities of daily living (preserved), *BMI* body mass index, *EFS* Edmonton Frail Scale total score, *HAM-D* Hamilton depression rating scale, *IADL* instrumental activities of daily living (preserved), *MMSE* mini-mental state examination, *MNA* mini nutritional assessment, *MoCA* Montreal Cognitive Assessment, *SF-36* short form health survey 36 (SF-36 mean score was obtained by calculating the arithmetic mean of the scores relative to the 9 items of SF-36), *SPPB* short physical performance battery, *VAT* visceral adipose tissue.

^a^Mann-Whitney U test.

^b^Chi Square test.

^c^Four-way ANCOVA with frailty status, sex, diabetes, and smoking as factors, age and BMI as covariates. The analysis was conducted on log-transformed amino acid concentrations to normalize the data distribution. Log-transformed values are reported as Suppl. Table A in Zenodo repository (10.5281/zenodo.10669703).

MMSE and MoCA scores did not correlate with age in either non-frail (r = 0.214, p = 0.075 and r = −0.013, p = 0.918, respectively) or frail group (r = −0.180, p = 0.220 and r = −0.236, p = 0.106, respectively), indicating that the difference in MoCA score between non-frail and frail groups was not attributable to the older age of frail subjects.

### Serum levels of D-serine and D-/Total serine ratio correlate with EFS score

We first investigated whether the serum levels of amino acids were different between frail and non-frail groups adjusting for the effect of the potential confounders. ANCOVA showed no between-group differences in D-serine, L-serine or any of the other amino acids level (Table 1B).

**Fig. 1:**
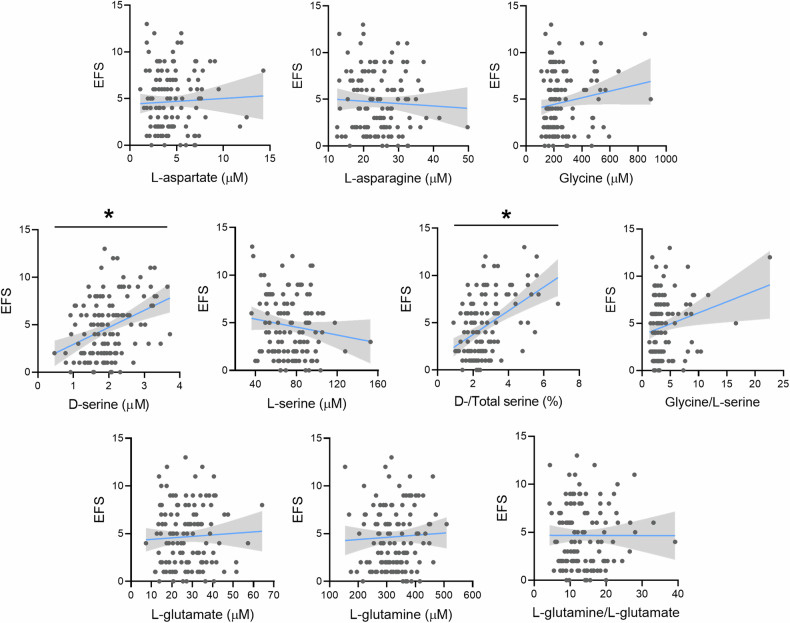
Correlations between the serum levels of amino acids and Edmonton Frailty Scale (EFS) total score in the whole elderly cohort. Blue lines and gray shadows represent the best-fit line and its 95% CI, respectively. *p < 0.05, age- and sex-adjusted partial correlation.

To further address this issue, we measured the partial correlation between the quantitative EFS score and the serum concentrations of amino acids, adjusting for age and sex. We found a significant mild positive correlation of EFS with serum D-serine (r = 0.197, p = 0.032) and D-/Total serine ratio (r = 0.213, p = 0.020), but not with the other amino acids (Fig. 1 and Suppl. Table 1) (Table 2).

**Table 2:** Multiple linear regression models for EFS prediction, including clinical variables and serum D-serine (model 1) or D-/Total serine ratio (model 2) as predictors.

	β	SE	Std β	p
Model 1: D-Serine and clinical features as predictors of EFS				
Constant	3.087	3.833		0.422
Age (years)	0.115	0.036	0.255	**0.002**
Male sex	0.214	0.649	0.029	0.742
MNA	−0.159	0.068	−0.167	**0.022**
Handgrip (kg)	−0.087	0.034	−0.260	**0.011**
BADL	0.095	0.269	0.031	0.723
IADL	−0.170	0.168	−0.101	0.313
HAM-D	0.021	0.030	0.042	0.477
MoCA	−0.144	0.063	−0.151	**0.025**
Number of drugs	0.170	0.056	0.215	**0.003**
D-serine (µM)	0.704	0.321	0.136	**0.031**
Model 2: D-/Total serine and clinical features as predictors of EFS				
Constant	4.635	3.822		0.228
Age (years)	0.102	0.038	0.226	**0.008**
Male sex	0.201	0.645	0.027	0.756
MNA	−0.198	0.069	−0.208	**0.005**
Handgrip (kg)	−0.083	0.034	−0.248	**0.015**
BADL	0.065	0.268	0.021	0.809
IADL	−0.125	0.168	−0.075	0.458
HAM-D	0.017	0.030	0.033	0.574
MoCA	−0.140	0.063	−0.147	**0.029**
Number of drugs	0.167	0.056	0.212	**0.003**
D-/Total serine (%)	0.553	0.228	0.162	**0.017**

Complete clinical data were available for n = 110 subjects. Significant *p*-values are shown in bold.

*BADL* basic activities of daily living (preserved), *EFS* Edmonton Frail Scale, *IADL* instrumental activities of daily living (preserved), *HAM-D* Hamilton Depression Rating Scale, *MNA* Mini Nutritional Assessment, *MoCA* Montreal Cognitive Assessment, *SE* standard error of β, *Std β* standardized β coefficient.

### Correlation of serum levels of D-serine and D-/Total serine ratio with demographic and clinical features

We also investigated whether diabetes, obesity (BMI, VAT), sarcopenia (SMI), and cigarette smoking affected the serum concentration of amino acids. Diabetic subjects showed higher levels of L-asparagine, L-serine, L-glutamate, L-glutamine/L-glutamate ratio, and lower glycine/L-serine ratio than non-diabetic participants (Suppl. Table 2). After adjustment for age and sex, L-glutamate and L-glutamine/L-glutamate correlated with (i) BMI and VAT in both non-frail and frail participants; (ii) SMI only in the non-frail group (Suppl. Table 3). Current and former smokers had reduced L-glutamine/L-glutamate ratio compared to never smokers (Suppl. Table 4).

**Fig. 2:**
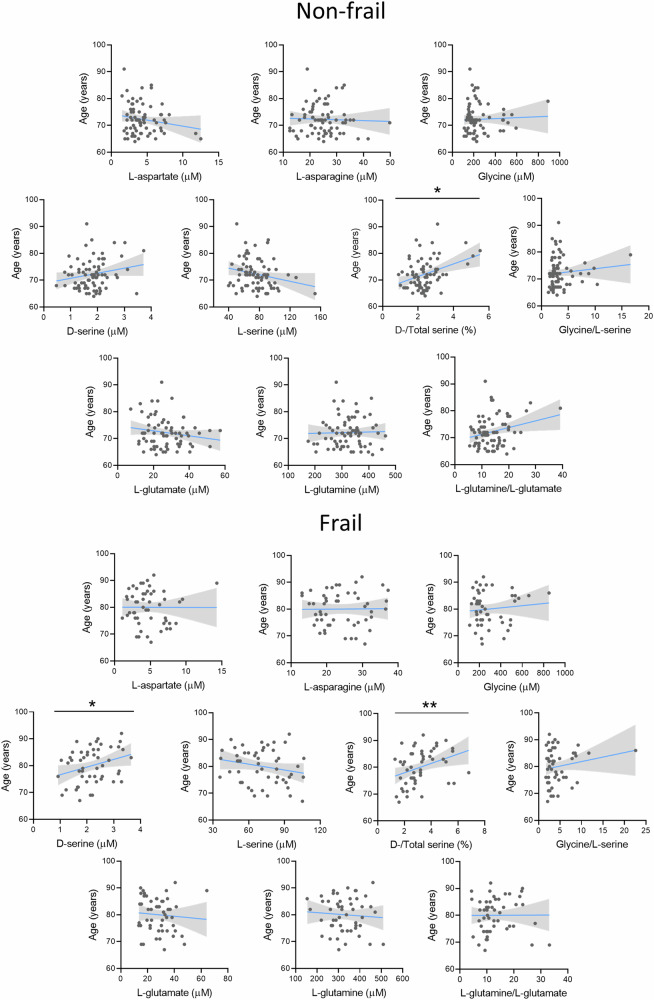
Correlations between the serum amino acids concentrations and age in elderly cohort stratified in frail and non-frail groups according to EFS. Blue lines and gray shadows represent the best fit line and its 95% CI, respectively. *p < 0.05; **p < 0.01, Spearman’s correlation test.

Serum D-serine correlated with age in the frail (r = 0.299, p = 0.033) but not in non-frail group, while D-/Total serine ratio correlated with age both in non-frail (r = 0.278, p = 0.017) and frail subjects (r = 0.415, p = 0.002) (Fig. 2 and Suppl. Table 5).

### Serum levels of D-serine and D-/Total serine ratio are independent predictors of frailty

Furthermore, to assess whether serum levels of D-serine and D-/Total serine ratio are independently associated with frailty, we performed multiple linear regression models using the quantitative EFS score as dependent variable and the known clinical predictors of EFS [9], added to the individual amino acids concentrations, as predictors. Interestingly, increased levels of D-serine and D-/Total serine ratio, but not the other amino acids, resulted to be independent predictors of EFS score, along with older age and worse nutritional status, handgrip, global cognition, and higher number of drugs (Table 2 and Suppl. Tables B–I). These findings highlight that an abnormally greater serum D-/Total serine ratio, used as an index of D-serine metabolism [47], along with blood D-serine concentrations, may represent a putative biochemical marker of frailty in elderly people.

### Increased serum glycine/L-serine and D-/Total serine ratios correlate with worse global cognition in frail elderly subjects

**Fig. 3:**
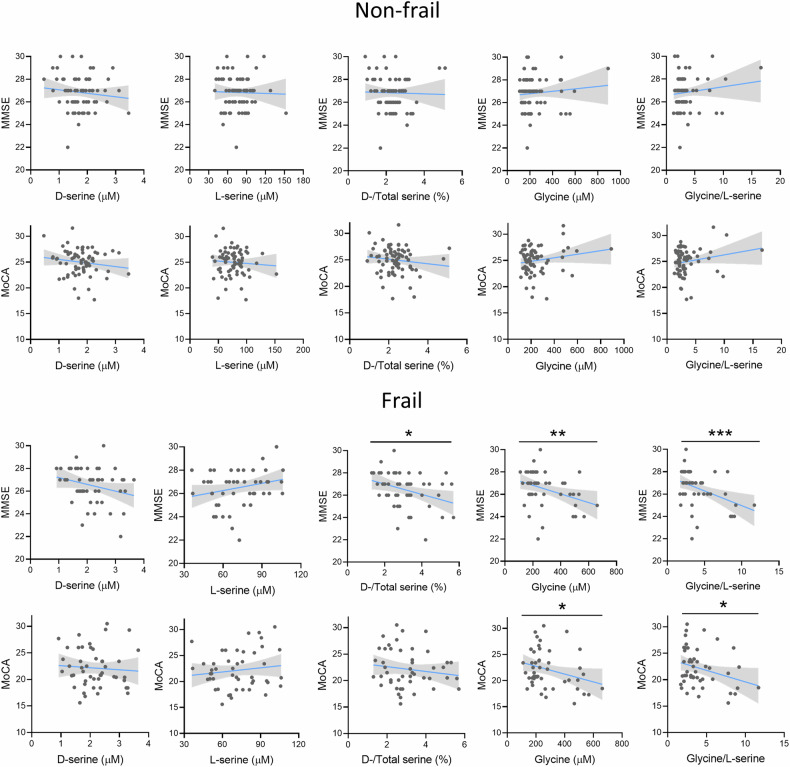
Correlations between the serum amino acids concentrations and measures of global cognition in elderly cohort stratified in frail and non-frail groups according to EFS. Blue lines and gray shadows represent the best-fit line and its 95% CI, respectively. *p < 0.05; **p < 0.01, age and sex-adjusted partial correlations. Abbreviations: MMSE Mini-Mental State Examination, MoCA Montreal Cognitive Assessment.

Next, we investigated whether serum D-serine, D-/Total serine ratio, and the other amino acids were associated with one or more of the frailty domains which concur to determine the EFS score. Notably, we found negative partial correlations between (i) glycine, glycine/L-serine ratio, D-/Total serine ratio, and MMSE; (ii) glycine, glycine/L-serine ratio, and MoCA score in the frail but not in the non-frail subjects (Fig. 3). The other amino acids did not correlate with cognitive measures (Suppl. Fig. 1 and Suppl. Table J). Moreover, L-asparagine and L-glutamine correlated negatively with HAM-D score, while glycine levels and glycine/L-serine ratio increased with worse depressive symptoms in frail but not in non-frail subjects (Table 3). There were no significant correlations between the serum amino acids and the other frailty domains (Suppl. Tables K–L).

**Table 3:** Correlations between the serum levels of amino acids and HAM-D score in the elderly cohort stratified in non-frail and frail groups according to EFS.

	L-aspartate		L-asparagine		Glycine		D-serine		L-serine		Glycine/L-serine		D-/Total serine		L-glutamate		L-glutamine		L-glutamine/L-glutamate	
	r	p	r	p	r	p	r	p	r	p	r	p	r	p	r	p	r	p	r	p
Non-frail	−0.073	0.549	−0.019	0.876	−0.004	0.976	−0.026	0.833	−0.030	0.804	0.049	0.687	0.013	0.918	0.057	0.638	0.014	0.906	−0.063	0.603
Frail	0.012	0.938	−0.403	**0.007**	0.389	**0.009**	−0.052	0.736	−0.241	0.116	0.526	**<0.001**	0.131	0.395	0.067	0.667	−0.347	**0.021**	−0.255	0.094

HAM-D score was available for 72 non-frail and 46 frail participants. Correlation coefficients and p-values refer to age and sex-adjusted partial correlations. Significant *p*-values are shown in bold.

Overall, these findings indicate that dysregulated blood glycine/L-serine and D-/Total serine ratios may represent metabolic biomarkers of cognitive impairment and depressive symptoms in frail older subjects.

### Serum D-serine and D/Total serine do not correlate with physical frailty phenotype

To further evaluate the relationship between serum amino acids and frailty, we stratified the elderly cohort according to Fried’s frailty phenotype [8]. Based on these criteria, 22 subjects were classified as non-frail, 51 as pre-frail, and 52 as frail. After adjusting for the effect of potential confounders, there were no significant differences in the serum concentrations of the tested amino acids between the 3 groups (Suppl. Table 6). To better assess whether the serum levels of these metabolites may associate with physical frailty phenotype, we performed multinomial logistic regression models with Fried phenotype as dependent variable and age, sex, and the individual amino acids concentrations as predictors. Remarkably, we found that neither the levels of D-serine, nor those of the other amino acids, were associated with physical frailty or pre-frailty status (Suppl. Table 7 and Suppl. Table M).

Taken together, these findings suggest that the blood levels of D-serine and D-/Total serine ratio are not associated with the physical domain of frailty in elderly individuals.

### The correlations between serum D-serine, D-/Total serine ratio, and EFS are driven by female sex

The demographic and clinical features of the elderly cohort stratified by sex are reported in Suppl. Table 8A. Females showed a worse impairment in physical and quality of life domains compared to males. Although the difference was not statistically significant, females also had a higher EFS score than males. The serum concentrations of amino acids were similar between females and males (Suppl. Table 8B). D-serine, D/Total serine and glycine/L-serine ratios positively correlated with age in both sexes, while L-serine selectively decreased with older age only in males (Suppl. Table N). Consistent with these HPLC data, we found that the positive correlation between D-serine, D-/Total serine, and EFS score observed in the whole cohort (Fig. 1) was mainly driven by female sex (Suppl. Table O). The multiple linear regression models adjusted for the clinical predictors of EFS showed that the levels of D-serine and D-/Total serine, but not the other amino acids, were independent predictors of EFS score in females (β = 0.989, p = 0.008, and β = 0.748, p = 0.007, respectively) but not in males (Suppl. Table P).

### Increased serum glycine/L-serine and D-/Total serine ratios correlate with worse global cognition and quality of life in a sex-dependent manner

We found a negative correlation between D-serine (r = −0.222, p = 0.035), D-/Total serine (r = −0.289, p = 0.006), glycine/L-serine (r = −0.299, p = 0.004) and MMSE score in females but not in males. Moreover, D-/Total serine negatively correlated with MoCA (r = −0.235, p = 0.025), SF-36 General Health (r = −0244, p = 0.021) and SPPB total score (r = −0.209, p = 0.043) in females but not in males. Finally, L-asparagine and L-glutamine correlated negatively with HAM-D score, while glycine and glycine/L-serine ratio increased with worse depressive symptoms in females but not in males (Suppl. Tables Q–Y).

## Discussion

Compelling studies have shown that changes in the cerebrospinal fluid (CSF) and blood levels of amino acids acting on the glutamatergic N-methyl-D-aspartate receptor (NMDAR) represent a neurochemical signature in various neuropathologies. These include psychiatric conditions such as schizophrenia [47, 48], major depression [49], and a wide spectrum of neurological diseases, including Alzheimer’s disease (AD) [45, 50, 51], frontotemporal dementia [52], Parkinson’s disease (PD) [43, 44, 53, 54], amyotrophic lateral sclerosis [55, 56], mild cognitive impairment [57, 58], multiple sclerosis [59, 60] and traumatic brain injury [61]. Surprisingly, no investigation so far specifically addressed the relationship between these neuroactive molecules and frailty phenotypes, including those related to cognitive decline and depression. Here, we sought to fill this gap by investigating the endogenous levels of D-serine, glycine, and the other amino acids acting on glutamatergic neurotransmission in a well-characterized cohort of older subjects encompassing the entire continuum existing between fit and frail aging. Overall, our biochemical determinations suggest that disrupted systemic D-serine homeostasis may represent a potential predictive biomarker of frailty, while increased serum glycine/L-serine and D-/Total serine ratios could be associated with cognitive decline and depression in frail elderly individuals.

Previous blood metabolomics studies identified several metabolites associated with frailty, belonging to redox homeostasis, inflammation, amino acids, purine metabolism, urea and tricarboxylic acid cycles, and sugar metabolism pathways [13]. Among the amino acids identified as dysregulated, glutamate metabolism was found to be affected in frail compared to non-frail subjects [15–18, 21]. In light of this finding, and given the close relationship linking frailty with cognitive decline [11, 26, 62], we investigated whether the serum levels of amino acids acting on glutamatergic NMDAR and their precursors could predict frailty status, and specifically its cognitive domain, in elderly adults. Interestingly, we found that serum D-serine is an independent predictor of the EFS score. D-serine is synthesized by serine racemase (SR) [63] starting from its L-enantiomer and then degraded through D-amino acid oxidase (DAO) activity [64, 65]. Once released in the forebrain, D-serine act as an obligatory co-agonist at the glycine modulatory site on GluN1 subunit of NMDAR, a ionotropic glutamatergic receptor playing a key role in sensorimotor gating, synaptic plasticity and cognitive functions [66]. Despite a few reports suggested that circulating blood D-serine concentrations slightly decrease [66] or remain unchanged [44, 46, 67] during healthy aging, recent studies found a positive correlation between serum D-serine and age in patients affected by AD and PD [44, 46].

Our observations showing that D-serine and D-/Total serine ratio significantly increase with aging in frail but not in non-frail controls suggests that a dysregulation of blood D-serine homeostasis may represent a common ageing-related metabolic variation across different neuropathologies.

While EFS was conceived to evaluate frailty through a multidimensional approach, the Fried’s frailty phenotype is a widely used tool able to capture the physical domain of frailty [7]. Notably, we failed to find any association between D-serine or the other amino acids levels and frailty phenotype. Therefore, we argue that D-serine may not mirror all the components of frailty syndrome, but could instead represent a biochemical fingerprint of its cognitive domain. Consistent with this view, D-serine has recently been proposed as an early gender-related biomarker of AD since its serum concentrations correlated with cognitive deterioration in female patients [46, 51]. However, other Authors failed to confirm significant changes of CSF and blood D-Ser levels in the whole AD clinical spectrum [45, 68]. Interestingly, a recent clinical-pathological study showed that Aβ and tau brain deposition and frailty have a synergistic impact in determining the onset of dementia [62]. This finding, considered together with (i) the previous studies linking increased D-serine with AD-related pathology and cognitive decline [50, 51, 69] and (ii) the ability of D-serine to diffuse across the blood-brain barrier [70], suggests that blood levels of this D-amino acid could be adopted as a metabolic marker to identify older adults at higher risk of conversion to dementia.

Notably, the stratification of our elderly cohort by sex disclosed that the correlation between serum D-serine, EFS score, and global cognition was mainly driven by females. In agreement with this view, recent investigations showed increased D-/Total Ser ratio in the human post-mortem hippocampus and serum of AD female patients compared to healthy females [46, 69]. Similarly, we recently found a significant increase of serum D-serine in PD female, but not in male patients, compared to healthy controls [44]. These findings suggest that a dysregulation of blood D-serine may reflect the occurrence of different neuropathologies in a sex-dependent manner. Considering the neuroprotective role played by estrogens and the compelling evidence that estrogens loss after menopause can accelerate the effect of aging on cognitive functions [71], we speculate that the link between increased systemic D-serine levels and cognitive decline may be mediated, at least in part, by the reduced estrogens levels which characterize females aging. However, further studies on larger elderly cohorts are needed to address this outstanding issue.

Remarkably, we also found that higher serum glycine concentrations and glycine/L-serine ratio correlated with worse cognitive function and depressive symptoms in the frail but not in the non-frail group. Similarly to D-serine, glycine binds the GluN1 subunit of NMDAR and acts as a major obligatory co-agonist [72]. However, in other central nervous system (CNS) regions, glycine also regulates inhibitory neurotransmission via glycine receptors (GlyR) [73]. Considering that a dysfunctional glycinergic transmission has been implicated in the physiopathology of cognitive decline and depression [72, 73], the correlation between blood glycine levels, cognitive performance, and depressive symptoms may mirror an altered metabolism of this amino acid in the CNS of frail subjects.

Despite our biochemical data are highly intriguing, the findings of the present study should be interpreted cautiously, bearing in mind that (i) the assessment of AD and other dementia-related biomarkers was not included in this study, thus preventing any inference linking the serum amino acid levels and the presence of concomitant neurodegenerative diseases; (ii) frailty is characterized by a decline in the function of multiple organ systems, which may directly influence the serum concentration of D-serine, glycine and the other amino acids. Indeed, recent studies showed that blood D-serine levels correlate positively with biochemical renal parameters [67, 74–76], while various L-amino acids correlated with metabolic parameters such as liver enzymes, lipids, and blood glucose [67]. Moreover, blood glycine levels may be affected by physical exercise [77], regional adiposity [78], and bone mineral density [79].

Dietary intake and D-amino acids produced by the gut microbiota may also affect serine enantiomers metabolism [80, 81]. Gut commensal bacteria represent indeed a main source of several D-amino acids in mammals, including D-serine, and the impact of gut microbiota metabolism on blood levels of D-amino acids, peripheral organs, and the gut-brain axis function is currently a hot research topic [82]. For instance, D-glutamate synthesized by gut bacteria has been proposed to influence the NMDAR neurotransmission and cognitive function in AD patients [82]. This result support the hypothesis that gut microbiota plays a role in modulating the gut-brain axis through D-amino acids metabolism, and could therefore be a potential target of intervention in neurological and neuropsychiatric diseases [82, 83]. Further studies correlating the peripheral D-serine levels with the composition of gut microbiota in elderly frail individuals with and without neurological disorders are warranted.

Interestingly, studies in animal models showed that D-serine is detectable in multiple organs, including heart, pancreas, spleen, liver, kidney, lung and muscles [84, 85], and glutamatergic receptors play relevant functions in the modulation of physiological processes in several peripheral tissues [86]. Of note, recent studies showed that SR and NMDAR are highly expressed in human pancreatic islet β cells [87], and systemic D-serine administration modulates insulin secretion in a dose-dependent manner [88, 89]. Despite we found similar serum D-serine levels between diabetic and non-diabetic subjects, L-serine and L-glutamate were increased in diabetic compared to non-diabetic group, consistently with previous blood metabolomics evidence [16, 17]. In line with other studies [90], we also observed a positive correlation between serum L-glutamate concentration, BMI and visceral adiposity in both non-frail and frail participants. Although the biological mechanisms responsible for this association are still unclear, considering that glutamate signaling modulates the immune system [91] and that increased VAT promotes systemic inflammation [92], elevated blood L-glutamate levels could represent a metabolic signature underpinning the abnormal increase in oxidative stress and inflammation associated with obesity. Concurrently, our data suggest a correlation between serum L-glutamate concentration and SMI. This is in line with previous investigations showing that glutamate is crucial in maintaining the homeostasis of energy metabolism in skeletal muscle [93]. Surprisingly, this relationship was observed in the non-frail but not in the frail group, suggesting that different biological pathways may modulate the maintenance of skeletal muscle mass across healthy and frail aging. However, our results may be affected by the very low prevalence of subjects with SMI scores below the proposed cut-off to define sarcopenia [38] and therefore require validation in larger cohorts.

In the kidney, liver, and other peripheral organs, L-serine is rapidly interconverted with glycine in a single reaction catalyzed by serine hydroxymethyltransferase (SHMT) as part of the folate-mediated one‑carbon metabolism [94]. This direct relationship makes the serum glycine/L-serine ratio a reliable index of serine-glycine interconversion [95]. Consequently, the positive correlation of serum glycine/L-serine and D-/Total serine ratios with cognitive dysfunction and depressive symptoms observed in the frail group indicates that disturbed serine-glycine metabolism emerge as a peripheral proxy of brain functions decline in frail elderly populations.

Besides its neuroactive role, glycine primarily influences anti-oxidative reactions and immune system [94]. In agreement with this knowledge, glycine has been used to prevent tissue injury, enhance anti-oxidative capacity, improve immunity, and treat metabolic disorders in obesity, diabetes and various inflammatory diseases [96]. Thus, consistently with the reported “geroprotective” effects of glycine, we cannot rule out that the negative correlation of this amino acid and the glycine/L-serine ratio with cognitive function in frail older individuals might represent an adaptive mechanism aimed at counteracting the systemic inflammation and metabolic dysfunctions that characterize frailty syndrome [12, 25], rather than being causally linked to cognitive impairment.

In the same way, we argue that systemic D-serine metabolism variation in frailty may represent a biochemical adaptation to CNS and multi-system deteriorations. In line with this idea, D-serine supplementation or treatment with DAO inhibitors significantly improved cognitive functions in animal models of aging [97], as well as in healthy subjects, PD, and schizophrenia patients [98–101].

Given that (i) altered CNS and peripheral homeostasis of the NMDAR-stimulating amino acids have been observed in multiple brain pathologies [102] and non-neurological disorders [67]; (ii) these amino acids are directly involved in a plethora of neuronal processes and metabolic pathways, we cannot assume that changes in their concentrations observed in this study are exclusive of frailty syndrome. Conversely, serine enantiomers and glycine metabolism variation may represent a common biochemical marker of brain dysfunctions across a broad spectrum of neurological and non-neurological conditions.

Our findings have practical implications for future clinical research. First, this study paves the way for further investigations evaluating the levels of the two enzymes implicated in glycine—L-serine and L-serine—D-serine interconversion (SHMT and DAO, respectively) as putative markers of brain function in frail elderly cohorts. In line with this, previous evidence showed that blood DAO levels increase during cognitive progression in AD [57] and amnestic mild cognitive impairment [102, 103]. Moreover, single nucleotide polymorphisms in the *DAOA* gene, which encodes for the DAO activator protein G72, have been associated with schizophrenia [104] and autism spectrum disorder [105]. On the other hand, SHMT may play neuroprotective roles in AD [106] and PD [107]. Second, our study lays the foundation for future metabolomics investigations aimed at comprehensively assessing the whole serine-glycine and other amino acids metabolism in frailty. Third, the use of D-/Total-serine and glycine/L-serine ratios instead of the single amino acids concentrations makes these indexes easily comparable between different analytical techniques, thus simplifying the translation of our approach to other research laboratories.

The strengths of our work include (i) the novelty of investigating NMDAR-related amino acids and their precursors in the serum of a well-characterized elderly cohort, including the entire clinical spectrum existing from fit to frail condition; (ii) the adjustment of statistical analyses with multiple potential confounding factors which may affect the serum amino acids concentration, such as diabetes, body composition and cigarette smoking [108]; (iii) the assessment of frailty with two different but complementary tools [7, 8].

However, we also acknowledge some limitations. First, the cross-sectional design and the clinical-biochemical correlations observed in the present study did not allow to define the cause-effect relationship between the serum changes in amino acids levels and the clinical phenotypes. Future longitudinal studies on larger elderly cohorts adopting a multidimensional approach, including the measurement of blood biomarkers mirroring brain (e.g., neurofilament light chain, Aβ42/Aβ40 ratio, phosphorylated tau [109]) and peripheral organs damage, as well as inflammation, are warranted to elucidate this issue. Second, the sex ratio was unbalanced with a higher prevalence of females, potentially biasing the analyses conducted after stratifying the cohort by sex. Third, the assessment of biochemical parameters of kidney and liver function was not included in the study protocol, thus preventing the adjustment of the analyses for the serum levels of creatinine, aspartate transaminase, and alanine transaminase, which correlate with the blood levels of D-serine and several L-amino acids, respectively [67]. However, the history of any kidney or liver disease or altered parameters of renal and hepatic function was strictly considered as exclusion criteria at the time of participants’ enrollment.

In conclusion, this study suggests that increased serum D-/Total serine and glycine/L-serine ratios may mirror worse cognitive decline and depressive symptoms in frail older subjects. The observation that D-serine correlates with frailty scores and global cognition in females but not in males suggest that this effect may also be modulated by sex-related biological factors.

### Supplementary information

Supplementary Figure 1

Supplementary Tables 1–8

Supplementary Tables A–W

## Data Availability

The dataset generated during the current study is available in the ZENODO repository (10.5281/zenodo.10669703).
