# Supplementary material for: Serum dysregulation of serine and glycine metabolism as predictive biomarker for cognitive decline in frail elderly subjects
Source: Transl Psychiatry. 2024 Jul 9;14:281. doi: 10.1038/s41398-024-02991-z (PMC11233661; doi:10.1038/s41398-024-02991-z)
Supplement: Supplementary file 1 — Supplementary Figure 1 [file 41398_2024_2991_MOESM1_ESM.docx]

**
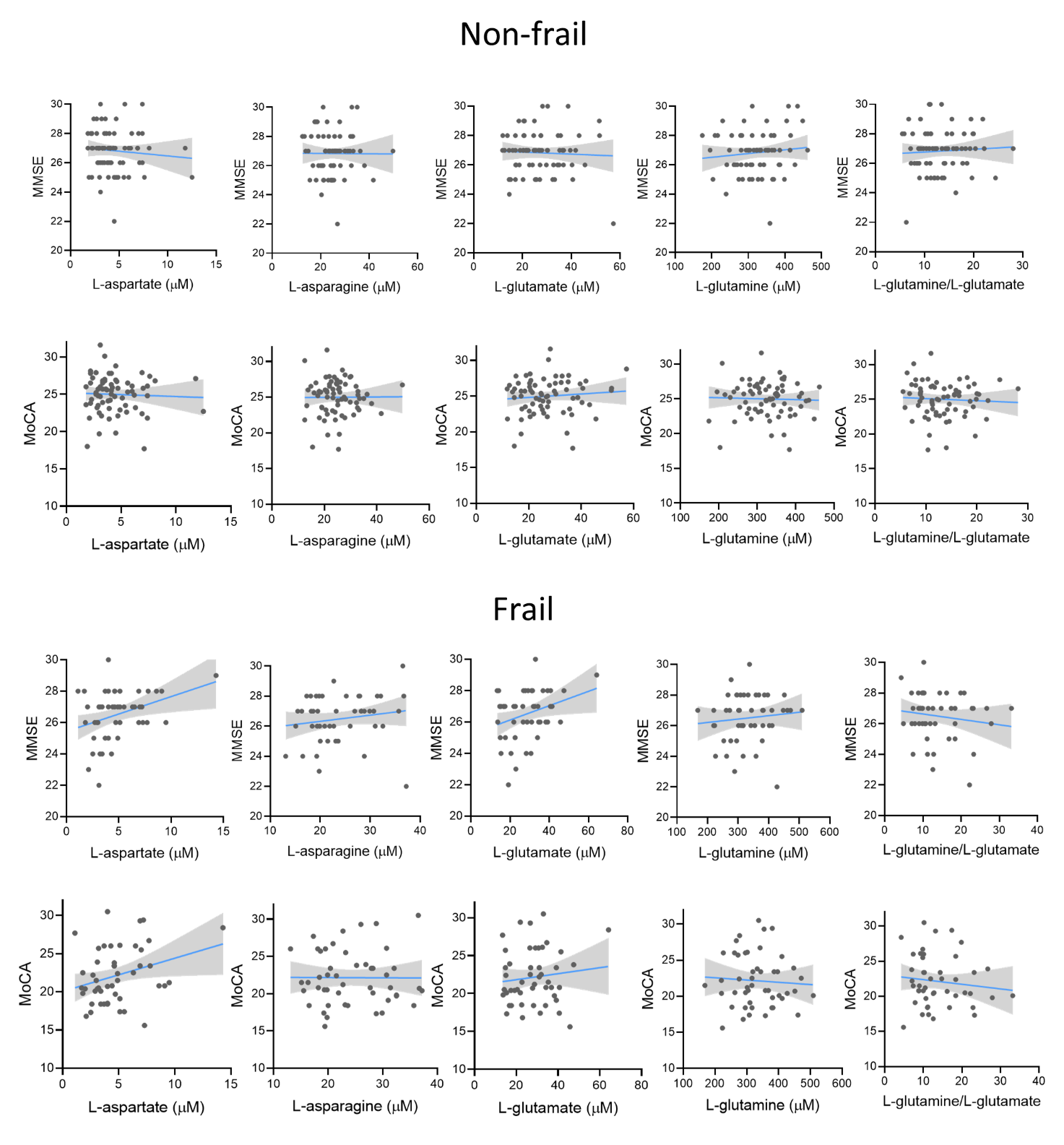
Supplementary Figure 1.** Correlations between the serum L-aspartate, L-asparagine, L-glutamate, L-glutamine concentration, L-glutamine/L-glutamate ratio, and measures of global cognition in elderly cohort stratified in frail and non-frail groups according to EFS. Blue lines and grey shadows represent the best fit line and its 95% CI, respectively. * p < 0.05; **p < 0.01, age and sex-adjusted partial correlations. Abbreviations: MMSE, Mini-Mental State Examination; MoCA, Montreal Cognitive Assessment.
