# Supplementary material for: Serum dysregulation of serine and glycine metabolism as predictive biomarker for cognitive decline in frail elderly subjects
Source: Transl Psychiatry. 2024 Jul 9;14:281. doi: 10.1038/s41398-024-02991-z (PMC11233661; doi:10.1038/s41398-024-02991-z)
Supplement: Supplementary file 2 — Supplementary Tables 1–8 [file 41398_2024_2991_MOESM2_ESM.docx]

**Serum dysregulation of serine and glycine metabolism as predictive biomarker for cognitive decline in frail elderly subjects**

Alberto Imarisio, MD, Isar Yahyavi, PhD, Clara Gasparri, MSc, Amber Hassan, PhD, Micol Avenali, MD, PhD, Anna Di Maio, MSc, Gabriele Buongarzone, MD, Caterina Galandra, PhD, Marta Picascia, PsyD, Asia Filosa, MSc, Maria Cristina Monti, PhD, Claudio Pacchetti, MD, Francesco Errico, PhD, Mariangela Rondanelli, MD, PhD, Alessandro Usiello, PhD, Enza Maria Valente, MD, PhD

**Supplementary Tables 1-8**

Please note that additional supplementary tables (A-W) and the dataset generated in this study will be available as raw data in the Zenodo repository (DOI: 10.5281/zenodo.10669703).

**Supplementary Table 1.** Correlations between the serum levels of amino acids and EFS total score in elderly cohort.

|  | **Total (n = 125)** | |
| --- | --- | --- |
|  | **r** | **p^a^** |
| **L-aspartate (μM)** | 0.047 | 0.609 |
| **L-asparagine (μM)** | -0.046 | 0.620 |
| **Glycine (μM)** | 0.113 | 0.221 |
| **D-serine (μM)** | 0.197 | **0.032** |
| **L-serine (μM)** | 0.036 | 0.707 |
| **Glycine/L-serine** | 0.126 | 0.172 |
| **D-/Total serine (%)** | 0.213 | **0.020** |
| **L-glutamate (μM)** | 0.078 | 0.400 |
| **L-glutamine (μM)** | 0.073 | 0.430 |
| **L-glutamine/L-glutamate** | -0.050 | 0.588 |

^a^ partial correlation adjusted for age, sex, diabetes, BMI and smoking.

**Supplementary Table 2.** Serum amino acids levels in elderly cohort stratified in subjects with and without type 2 diabetes mellitus. Data are shown as median (IQR).

|  | **Non-diabetic (n = 104)** | **Diabetic (n = 21)** | **p^a^** |
| --- | --- | --- | --- |
| **L-aspartate (μM)** | 3.9 (2.9-5.2) | 4.7 (3.6-7.3) | 0.056 |
| **L-asparagine (μM)** | 23.4 (19.8-27.7) | 31.1 (21.0-34.2) | **0.004** |
| **Glycine (μM)** | 215.6 (173.9-313.6) | 198.9 (176.2-225.2) | 0.126 |
| **D-serine (μM)** | 1.9 (1.6-2.2) | 1.8 (1.5-2.6) | 0.828 |
| **L-serine (μM)** | 71.3 (59.0-85.6) | 83.4 (60.8-94.8) | **0.015** |
| **Glycine/L-serine** | 3.0 (2.3-4.4) | 2.2 (2.0-3.3) | **0.010** |
| **D-/Total serine (%)** | 2.5 (2.0-3.2) | 2.5 (1.7-3.3) | 0.104 |
| **L-glutamate (μM)** | 25.0 (17.8-31.8) | 35.6 (27.8-40.0) | **0.003** |
| **L-glutamine (μM)** | 317.7 (279.3-368.5) | 337.1 (293.3-398.5) | 0.217 |
| **L-glutamine/L-glutamate** | 12.7 (9.9-18.0) | 10.2 (8.6-12.7) | **0.033** |

^a^ Two-way ANCOVA on log-transformed amino acids concentrations with diabetes and sex as factors, age as covariate

**Supplementary Table 3.** Correlations between the serum levels of amino acids and BMI, VAT and SMI in the elderly cohort. Data were available for 74 non-frail and 51 frail (BMI, SMI) and 73 frail and 51 frail subjects (VAT).

|  | **BMI** | | | | **VAT** | | | | **SMI** | | | |
| --- | --- | --- | --- | --- | --- | --- | --- | --- | --- | --- | --- | --- |
|  | **Non-frail** | | **Frail** | | **Non-frail** | | **Frail** | | **Non-frail** | | **Frail** | |
|  | **r** | **p** | **r** | **p** | **r** | **p** | **r** | **r** | **r** | **p** | **r** | **p** |
| **L-aspartate (μM)** | 0.120 | 0.316 | 0.003 | 0.984 | 0.145 | 0.229 | 0.136 | 0.352 | 0.068 | 0.573 | -0.127 | 0.386 |
| **L-asparagine (μM)** | -0.111 | 0.352 | -0.048 | 0.743 | -0.040 | 0.743 | -0.050 | 0.732 | 0.001 | 0.995 | 0.094 | 0.521 |
| **Glycine (μM)** | -0.055 | 0.645 | -0.101 | 0.488 | -0.025 | 0.839 | -0.055 | 0.708 | -0.054 | 0.649 | -0.054 | 0.710 |
| **D-serine (μM)** | 0.066 | 0.584 | -0.171 | 0.241 | 0.081 | 0.502 | -0.027 | 0.852 | 0.076 | 0.527 | -0.189 | 0.192 |
| **L-serine (μM)** | -0.044 | 0.712 | 0.040 | 0.786 | -0.060 | 0.621 | 0.140 | 0.336 | 0.044 | 0.714 | -0.015 | 0.918 |
| **Glycine/L-serine** | -0.022 | 0.855 | -0.058 | 0.691 | 0.014 | 0.906 | -0.057 | 0.696 | -0.058 | 0.626 | 0.019 | 0.896 |
| **D-/Total serine (%)** | 0.091 | 0.448 | -0.155 | 0.289 | 0.120 | 0.319 | -0.142 | 0.331 | 0.054 | 0.650 | -0.116 | 0.427 |
| **L-glutamate (μM)** | 0.288 | **0.014** | 0.268 | 0.063 | 0.261 | **0.028** | 0.430 | **0.002** | 0.328 | **0.005** | 0.022 | 0.879 |
| **L-glutamine (μM)** | -0.123 | -0.304 | -0.263 | 0.068 | -0.128 | 0.287 | -0.135 | 0.355 | 0.092 | 0.442 | -0.237 | 0.101 |
| **L-glutamine/L-glutamate** | -0.290 | **0.013** | **-0.385** | **0.006** | -0.322 | **0.006** | -0.424 | **0.002** | -0.233 | **0.049** | -0.161 | 0.268 |

**Abbreviations:** BMI, body mass index; SMI, skeletal muscle index; VAT, visceral adipose tissue. p-values refer to age and sex-adjusted partial correlations.

|  | **Never smokers (n = 88)** | **Former smokers (n = 22)** | **Current smokers (n = 15)** | **p^a^** |
| --- | --- | --- | --- | --- |
| **L-aspartate (μM)** | 4.0 (2.9-5.4) | 4.2 (3.4-5.4) | 3.8 (2.9-7.0) | 0.798 |
| **L-asparagine (μM)** | 24.8 (19.9-28.1) | 22.9 (19.7-30.1) | 23.2 (15.1-29.9) | 0.376 |
| **Glycine (μM)** | 207.3 (171.6-286.2) | 222.8 (175.6-356.4) | 200.3 (186.9-288.6) | 0.583 |
| **D-serine (μM)** | 1.9 (1.5-2.3) | 1.9 (1.6-2.7) | 1.9 (1.6-2.1) | 0.844 |
| **L-serine (μM)** | 72.1 (60.6-88.8) | 71.5 (58.8-85.8) | 75.8 (59.8-90.7) | 0.802 |
| **Glycine/L-serine** | 2.9 (2.2-4.1) | 2.9 (2.1-5.9) | 2.9 (2.1-3.3) | 0.659 |
| **D-/Total serine (%)** | 2.5 (2.0-3.2) | 2.7 (2.0-3.8) | 2.3 (2.1-3.3) | 0.674 |
| **L-glutamate (μM)** | 25.4 (17.4-32.3) | 27.7 (22.3-34.8) | 34.9 (22.8-40.3) | 0.067 |
| **L-glutamine (μM)** | 332.8 (286.6-427.8) | 298.0 (265.4-332.6) | 342.5 (280.5-396.7) | 0.097 |
| **L-glutamine/L-glutamate** | 13.0 (10.5-17.6) | 10.2 (8.9-13.7)^b^ | 9.9 (7.4-14.8)^b^ | **0.006** |

**Supplementary Table 4.** Serum amino acids levels in elderly cohort stratified according to cigarette smoking status. Data are shown as median (IQR).

^a^ Two-way ANCOVA on log-transformed amino acids concentrations with smoking status and sex as factors, age as covariate

^b^ compared with never smokers, p < 0.05 (Bonferroni corrected)

**Supplementary Table 5.** Correlations between the serum concentration of amino acids and age in elderly cohort stratified by frailty status according to EFS score.

|  | **Non-frail (n = 74 )** | | **Frail (n = 51)** | | **Total (n = 125)** | |
| --- | --- | --- | --- | --- | --- | --- |
|  | **Rho** | **p** | **Rho** | **p** | **Rho** | **p** |
| **L-aspartate (μM)** | -0.091 | 0.442 | -0.098 | 0.494 | -0.045 | 0.618 |
| **L-asparagine (μM)** | -0.061 | 0.606 | 0.026 | 0.858 | -0.066 | 0.466 |
| **Glycine (μM)** | 0.017 | 0.884 | 0.039 | 0.785 | 0.090 | 0.320 |
| **D-serine (μM)** | 0.142 | 0.228 | 0.299 | **0.033** | 0.330 | **< 0.001** |
| **L-serine (μM)** | -0.225 | 0.054 | -0.205 | 0.149 | -0.240 | **0.007** |
| **Glycine/L-serine** | 0.192 | 0.101 | 0.126 | 0.380 | 0.243 | **0.006** |
| **D-/Total serine (%)** | 0.278 | **0.017** | 0.415 | **0.002** | 0.468 | **< 0.001** |
| **L-glutamate (μM)** | -0.147 | 0.211 | -0.137 | 0.338 | -0.087 | 0.335 |
| **L-glutamine (μM)** | 0.008 | 0.945 | -0.044 | 0.761 | 0.002 | 0.985 |
| **L-glutamine/L-glutamate** | 0.158 | 0.179 | 0.163 | 0.254 | 0.102 | 0.257 |

**Abbreviations:** Rho, Spearman’s correlation coefficient.

**Supplementary Table 6.** Demographic features and serum amino acid levels in elderly cohort stratified according to Fried’s frailty phenotype. Data are shown as median (IQR) or absolute frequency (%) for continuous and categorical variables, respectively.

|  | **Non-frail (n = 22)** | **Pre-frail (n = 51)** | **Frail (n = 52)** | **Total (n = 125)** | **p** |
| --- | --- | --- | --- | --- | --- |
| **Age, years** | 68.0 (66.0-72.0) | 72.0 (69.0-76.0)^d^ | 82.0 (76.0-85.0)^e,f^ | 74.0 (69.5-81.0) | **< 0.001^a^** |
| **Female sex, n (%)** | 13 (59.1) | 37 (72.5) | 45 (86.5) | 95 (76.0) | **0.031^b^** |
| **L-aspartate (μM)** | 3.7 (3.1-5.2) | 4.0 (3.0-6.3) | 4.1 (2.9-5.5) | 4.0 (3.0-5.6) | 0.979^c^ |
| **L-asparagine (μM)** | 25.0 (20.5-27.8) | 24.1 (20.0-27.8) | 23.4 (19.1-29.6) | 24.1 (19.8-28.4) | 0.833^c^ |
| **Glycine (μM)** | 192.4 (144.8-229.0) | 202.3 (163.7-287.0) | 224.7 (186.1-330.0) | 208.8 (174.0-288.1) | 0.166^c^ |
| **D-serine (μM)** | 1.8 (1.5-2.1) | 1.8 (1.4-2.1) | 2.2 (1.7-2.6) | 1.9 (1.5-2.3) | 0.576^c^ |
| **L-serine (μM)** | 77.4 (64.0-89.4) | 71.7 (62.0-89.4) | 72.1 (53.4-84.8) | 72.5 (59.9-88.9) | 0.993^c^ |
| **Glycine/L-serine** | 2.3 (2.0-2.8) | 2.8 (2.2-4.0) | 3.3 (2.4-5.1) | 2.9 (2.2-4.1) | 0.234^c^ |
| **D-/Total serine (%)** | 2.3 (2.0-2.5) | 2.4 (1.9-2.9) | 2.8 (2.1-4.0) | 2.5 (2.0-3.2) | 0.633^c^ |
| **L-glutamate (μM)** | 25.3 (20.2-34.7) | 27.0 (18.9-34.5) | 26.7 (17.4-33.7) | 26.7 (19.0-34.2) | 0.970^c^ |
| **L-glutamine (μM)** | 345.4 (298.8-374.8) | 315.1 (280.5-358.4) | 317.6 (275.9-381.4) | 323.0 (280.5-370.3) | 0.771^c^ |
| **L-glutamine/L-glutamate** | 13.3 (10.3-16.5) | 12.0 (9.2-16.4) | 12.4 (9.9-18.2) | 12.0 (9.8-16.7) | 0.928^c^ |

^a^ Kruskal-Wallis test

^b^ Chi-square test

^c^ Four-way ANCOVA with frailty status, sex, diabetes and smoking as factors, age and BMI as covariates. The analysis was conducted on log-transformed amino acid concentrations to normalize the data distribution.

^d^ compared with non-frail, p < 0.05 (Bonferroni corrected)

^e^ compared with non-frail, p < 0.001 (Bonferroni corrected)

^f^ compared with pre-frail, p < 0.001 (Bonferroni corrected)

**Supplementary Table 7.** Multinomial logistic regression models for frailty phenotype prediction according to Fried criteria including A) D-serine and B) D-/Total serine as predictor. Non-frail status was set as reference category.

| **Model A** | | | | |
| --- | --- | --- | --- | --- |
|  | **β** | **SE** | **p** | **OR (95%CI)** |
| **Pre-frail** | | | | |
| Intercept | -15.813 | 5.351 | 0.003 |  |
| D-serine (µM) | 0.360 | 0.525 | 0.493 | 1.4 (0.5-4.0) |
| Age (years) | 0.221 | 0.076 | **0.004** | 1.2 (1.1-1.4) |
| Female sex | 0.601 | 0.594 | 0.311 | 1.8 (0.6-5.8) |
| **Frail** | | | | |
| Intercept | -31.930 | 6.135 | < 0.001 |  |
| D-serine (µM) | .784 | .604 | 0.194 | 2.1 (0.7-7.1) |
| Age (years) | .410 | .085 | **< 0.001** | 1.5 (1.3-1.8) |
| Female sex | 1.556 | .787 | 0.048 | 4.7 (1.0-22.2) |
| **Model B** | | | | |
| **Pre-frail** | | | | |
| Intercept | -15.341 | 5.308 | 0.004 |  |
| D-/Total serine (%) | 0.076 | 0.401 | 0.851 | 1.0 (0.4-2.3) |
| Age (years) | 0.221 | 0.078 | **0.005** | 1.2 (1.1-1.4) |
| Female sex | 0.528 | 0.582 | 0.364 | 1.6 (0.5-5.3) |
| **Frail** | | | | |
| Intercept | -30.913 | 6.084 | < 0.001 |  |
| D-/Total serine (%) | 0.295 | 0.426 | 0.490 | 1.3 (0.5-3.1) |
| Age (years) | 0.407 | 0.087 | **< 0.001** | 1.5 (1.3-1.8) |
| Female sex | 1.486 | 0.784 | 0.058 | 4.4 (0.9-20.5) |

**Supplementary Table 8**. Clinical and demographic features (A) and serum amino acid levels (B) of elderly cohort after stratification by sex. Data are shown as median (IQR) for continuous variables and as absolute frequency (%) for categorical variables. The total number of females (F) and males (M) for which data were availble is reported in the second column.

| **A) Demographic and clinical features** | | | | |
| --- | --- | --- | --- | --- |
|  | **N** | **Females** | **Males** | **p** |
| **Age, years** | 95 F, 30 M | 74.0 (71.0-82.0) | 72.0 (68.0-76.5) | 0.078^a^ |
| **MNA** | 94 F, 30 M | 23.5 (20.4-25.5) | 24.5 (22.5-25.5) | 0.196^a^ |
| **SPPB total score** | 95 F, 30 M | 7.0 (4.0-9.0) | 9.0 (7.0-10.3) | **0.009**^a^ |
| **Handgrip (kg)** | 95 F, 30 M | 19.0 (14.0-23.0) | 33.0 (25.0-40.5) | **< 0.001**^a^ |
| **SMI (kg/m^2^)** | 95 F, 29 M | 7.5 (7.0-8.3) | 8.6 (7.8-9.8) | **< 0.001^a^** |
| **MMSE** | 91 F, 27 M | 27.1 (26.0-27.7) | 26.3 (24.5-27.2) | 0.157^a^ |
| **MoCA** | 91 F, 27 M | 24.2 (21.5-26.1) | 24.1 (21.8-26.1) | 0.823^a^ |
| **BADL** | 90 F, 28 M | 6.0 (5.8-6.0) | 6.0 (6.0-6.0) | **0.047**^a^ |
| **IADL** | 90 F, 28 M | 8.0 (6.0-8.0) | 8.0 (6.0-8.0) | 0.491^a^ |
| **HAM-D** | 90 F, 28 M | 5.0 (2.0-10.2) | 5.5 (1.0-10.0) | 0.972^a^ |
| **SF-36 (mean score)** | 90 F, 27 M | 65.7 (49.8-76.3) | 77.9 (61.7-80.2) | **0.024**^a^ |
| **Number of drugs** | 93 F, 28 M | 4.0 (2.0-7.5) | 5.0 (3.0-8.0) | 0.921^a^ |
| **Type 2 diabetes, n (%)** | 95 F, 30 M | 14 (14.7) | 7 (23.3) | 0.272^b^ |
| **BMI (kg/m^2^)** | 95 F, 30 M | 27.8 (24.2-33.1) | 26.9 (23.6-31.9) | 0.640^a^ |
| **VAT (g)** | 95 F, 29 M | 893 (514-1522) | 1331 (928-2346) | **0.017^a^** |
| **Current smokers, n (%)** | 95 F, 30 M | 10 (10.5) | 5 (3.6) | 0.164^b^ |
| **Former smokers, n (%)** | 95 F, 30 M | 14 (14.7) | 8 (26.7) | 0.164^b^ |
| **Nevers smokers, n (%)** | 95 F, 30 M | 71 (74.7) | 17 (56.7) | 0.164^b^ |
| **EFS** | 95 F, 30 M | 5.0 (2.0-7.0) | 2.5 (1.8-5.3) | 0.080 |
| **B) Serum amino acid levels** | | | | |
|  | **N** | **Females** | **Males** | **p^c^** |
| **L-aspartate (μM)** | 95 F, 30 M | 4.1 (3.1-6.2) | 3.6 (2.9-5.2) | 0.307 |
| **L-asparagine (μM)** | 95 F, 30 M | 23.3 (19.3-27.9) | 25.5 (21.2-31.4) | 0.184 |
| **Glycine (μM)** | 95 F, 30 M | 215.9 (178.4-302.9) | 196.2 (157.4-229.2) | 0.123 |
| **D-serine (μM)** | 95 F, 30 M | 1.9 (1.5-2.3) | 2.0 (1.6-2.4) | 0.074 |
| **L-serine (μM)** | 95 F, 30 M | 73.7 (59.8-87.3) | 72.2 (62.1-90.7) | 0.980 |
| **Glycine/L-serine** | 95 F, 30 M | 3.0 (2.3-4.4) | 2.4 (2.0-3.4) | 0.380 |
| **D-/Total serine (%)** | 95 F, 30 M | 2.5 (2.0-3.2) | 2.6 (2.0-3.3) | 0.150 |
| **L-glutamate (μM)** | 95 F, 30 M | 26.7 (18.7-33.6) | 25.2 (19.1-34.9) | 0.523 |
| **L-glutamine (μM)** | 95 F, 30 M | 316.5 (276.4-365.0) | 339.9 (292.9-399.7) | 0.419 |
| **L-glutamine/L-glutamate** | 95 F, 30 M | 12.0 (9.9-16.4) | 13.4 (9.2-18.5) | 0.281 |

^a^ Mann-Whitney U test

^b^ Chi-square test

^c^ Three-way ANCOVA on log-transformed amino acid levels with sex, smoking and diabetes as factors, age and BMI as covariates

**Abbreviations**: BADL, basic activities of daily living (preserved); BMI, body mass index; EFS, Edmonton Frailty Scale total score; HAM-D, Hamilton depression rating scale; IADL, instrumental activities of daily living (preserved); MMSE, mini-mental state examination; MNA, mini nutritional assessment; MoCA, Montreal Cognitive Assessment; SF-36, Short Form Health Survey 36 (SF-36 mean score was obtained by calculating the arithmetic mean of the scores relative to the 9 items of SF-36); SPPB, short physical performance battery; VAT, visceral adipose tissue.
