# Supplementary material for: Serum dysregulation of serine and glycine metabolism as predictive biomarker for cognitive decline in frail elderly subjects
Source: Transl Psychiatry. 2024 Jul 9;14:281. doi: 10.1038/s41398-024-02991-z (PMC11233661; doi:10.1038/s41398-024-02991-z)
Supplement: Supplementary file 3 — Supplementary Tables A–W [file 41398_2024_2991_MOESM3_ESM.docx]

**Serum dysregulation of serine and glycine metabolism as predictive biomarker for cognitive decline in frail elderly subjects**

Alberto Imarisio, MD, Isar Yahyavi, PhD, Clara Gasparri, MSc, Amber Hassan, PhD, Micol Avenali, MD, PhD, Anna Di Maio, MSc, Gabriele Buongarzone, MD, Caterina Galandra, PhD, Marta Picascia, PsyD, Asia Filosa, MSc, Maria Cristina Monti, PhD, Claudio Pacchetti, MD, Francesco Errico, PhD, Mariangela Rondanelli, MD, PhD, Alessandro Usiello, PhD, Enza Maria Valente, MD, PhD

**Supplementary Tables A-W (to be uploaded as raw data in Zenodo repository).**

**Supplementary Table A.** Log-transformed serum amino acid concentrations in elderly cohort considered as a whole and after stratification by frailty status according to EFS. Data are shown as mean log (amino acid concentration) ± SD.

|  | **Total (n = 125)** | **Non frail (n = 74)** | **Frail (n = 51)** |
| --- | --- | --- | --- |
| **L-aspartate (μM)** | 0.6 ± 0.2 | 0.6 ± 0.2 | 0.6 ± 0.2 |
| **L-asparagine (μM)** | 1.4 ± 0.1 | 1.4 ± 0.l | 1.4 ± 0.1 |
| **Glycine (μM)** | 3.0 ± 0.2 | 2.3 ± 0.2 | 2.4 ± 0.2 |
| **D-serine (μM)** | 0.3 ± 0.3 | 0.2 ± 0.1 | 0.3 ± 0.1 |
| **L-serine (μM)** | 1.9 ± 0.1 | 1.9 ± 0.1 | 1.8 ± 0.1 |
| **Glycine/L-serine** | 0.5 ± 0.2 | 0.4 ± 0.2 | 0.5 ± 0.2 |
| **D-/Total serine (%)** | 0.4 ± 0.2 | 0.4 ± 0.1 | 0.5 ± 0.2 |
| **L-glutamate (μM)** | 1.4 ± 0.2 | 1.4 ± 0.2 | 1.4 ± 0.2 |
| **L-glutamine (μM)** | 2.5 ± 0.1 | 2.5 ± 0.1 | 2.5 ± 0.1 |
| **L-glutamine/L-glutamate** | 1.1 ± 0.2 | 1.1 ± 0.2 | 1.1 ± 0.2 |

**Supplementary Tables B-H.** Multiple linear regression models for EFS prediction including clinical variables and serum amino acids as predictors. Complete clinical data were available for n = 110 subjects.

*Suppl. Table B*

|  | **β** | **SE** | **Std β** | **p** |
| --- | --- | --- | --- | --- |
| Constant | 3.800 | 3.957 |  | 0.339 |
| Age (years) | 0.131 | 0.037 | 0.289 | **0.001** |
| Male sex | 0.431 | 0.657 | 0.058 | 0.513 |
| MNA | -0.168 | 0.072 | -0.177 | **0.021** |
| Handgrip (kg) | -0.092 | 0.035 | -0.273 | **0.009** |
| BADL | 0.115 | 0.275 | 0.038 | 0.676 |
| IADL | -0.174 | 0.172 | -0.104 | 0.315 |
| HAM-D | 0.017 | 0.030 | 0.035 | 0.567 |
| MoCA | -0.155 | 0.065 | -0.162 | **0.019** |
| Number of drugs | 0.166 | 0.057 | 0.210 | **0.005** |
| L-aspartate (µM) | -0.014 | 0.085 | -0.011 | 0.865 |

**Abbreviations:** BADL, basic activities of daily living (preserved); EFS, Edmonton Frailty Scale; IADL, instrumental activities of daily living (preserved); HAM-D, Hamilton Depression Rating Scale; MNA, Mini Nutritional Assessment; MoCA, Montreal Cognitive Assessment; SE, standard error of β; Std β, standardized β coefficient.

*Suppl. Table C*

|  | **β** | **SE** | **Std β** | **p** |
| --- | --- | --- | --- | --- |
| Constant | 4.401 | 4.056 |  | 0.281 |
| Age (years) | 0.128 | 0.037 | 0.283 | **0.001** |
| Male sex | 0.526 | 0.670 | 0.071 | 0.435 |
| MNA | -0.173 | 0.071 | -0.182 | **0.016** |
| Handgrip (kg) | -0.094 | 0.035 | -0.279 | **0.008** |
| BADL | 0.124 | 0.275 | 0.041 | 0.653 |
| IADL | -0.173 | 0.171 | -0.104 | 0.314 |
| HAM-D | 0.014 | 0.031 | 0.027 | 0.662 |
| MoCA | -0.151 | 0.065 | -0.158 | **0.022** |
| Number of drugs | 0.171 | 0.058 | 0.217 | **0.004** |
| L-asparagine (µM) | -0.019 | 0.030 | -0.041 | 0.523 |

**Abbreviations:** BADL, basic activities of daily living (preserved); EFS, Edmonton Frailty Scale; IADL, instrumental activities of daily living (preserved); HAM-D, Hamilton Depression Rating Scale; MNA, Mini Nutritional Assessment; MoCA, Montreal Cognitive Assessment; SE, standard error of β; Std β, standardized β coefficient.

*Suppl. Table D*

|  | **β** | **SE** | **Std β** | **p** |
| --- | --- | --- | --- | --- |
| Constant | 4.023 | 3.933 |  | 0.309 |
| Age (years) | 0.131 | 0.037 | 0.289 | **0.001** |
| Male sex | 0.416 | 0.656 | 0.056 | 0.527 |
| MNA | -0.169 | 0.070 | -0.177 | **0.018** |
| Handgrip (kg) | -0.093 | 0.035 | -0.277 | **0.008** |
| BADL | 0.155 | 0.281 | 0.051 | 0.582 |
| IADL | -0.199 | 0.174 | -0.119 | 0.258 |
| HAM-D | .0022 | 0.031 | 0.043 | 0.486 |
| MoCA | -0.157 | 0.064 | -0.164 | **0.016** |
| Number of drugs | 0.162 | 0.057 | 0.205 | **0.006** |
| Glycine (µM) | -0.001 | 0.001 | -0.042 | 0.497 |

**Abbreviations:** BADL, basic activities of daily living (preserved); EFS, Edmonton Frailty Scale; IADL, instrumental activities of daily living (preserved); HAM-D, Hamilton Depression Rating Scale; MNA, Mini Nutritional Assessment; MoCA, Montreal Cognitive Assessment; SE, standard error of β; Std β, standardized β coefficient.

*Suppl. Table E*

|  | **β** | **SE** | **Std β** | **p** |
| --- | --- | --- | --- | --- |
| Constant | 3.462 | 4.236 |  | 0.416 |
| Age (years) | 0.132 | 0.037 | 0.292 | **0.001** |
| Male sex | 0.425 | 0.660 | 0.057 | 0.521 |
| MNA | -0.162 | 0.072 | -0.171 | **0.027** |
| Handgrip (kg) | -0.092 | 0.035 | -0.273 | **0.009** |
| BADL | 0.117 | 0.277 | 0.038 | 0.672 |
| IADL | -0.179 | 0.173 | -0.107 | 0.304 |
| HAM-D | 0.018 | 0.031 | 0.036 | 0.555 |
| MoCA | -0.157 | 0.065 | -0.164 | **0.017** |
| Number of drugs | 0.164 | 0.057 | 0.208 | **0.005** |
| L-serine (µM) | 0.002 | 0.010 | 0.009 | 0.883 |

**Abbreviations:** BADL, basic activities of daily living (preserved); EFS, Edmonton Frailty Scale; IADL, instrumental activities of daily living (preserved); HAM-D, Hamilton Depression Rating Scale; MNA, Mini Nutritional Assessment; MoCA, Montreal Cognitive Assessment; SE, standard error of β; Std β, standardized β coefficient.

*Suppl. Table F*

|  | **β** | **SE** | **Std β** | **p** |
| --- | --- | --- | --- | --- |
| Constant | 3.669 | 3.905 |  | 0.350 |
| Age (years) | 0.133 | 0.037 | 0.293 | **< 0.001** |
| Male sex | 0.409 | 0.656 | 0.055 | 0.534 |
| MNA | -0.162 | 0.070 | -0.171 | **0.022** |
| Handgrip (kg) | -0.093 | 0.035 | -0.277 | **0.008** |
| BADL | 0.165 | 0.285 | 0.054 | 0.564 |
| IADL | -0.205 | 0.177 | -0.122 | 0.248 |
| HAM-D | 0.023 | 0.031 | 0.046 | 0.467 |
| MoCA | -0.156 | 0.064 | -0.164 | **0.017** |
| Number of drugs | 0.162 | 0.057 | 0.205 | **0.005** |
| Glycine/L-serine | -0.056 | 0.083 | -0.044 | 0.497 |

*Suppl. Table G*

|  | **β** | **SE** | **Std β** | **p** |
| --- | --- | --- | --- | --- |
| Constant | 3.589 | 3.893 |  | 0.359 |
| Age (years) | 0.131 | 0.036 | 0.289 | **0.001** |
| Male sex | 0.478 | 0.654 | 0.064 | 0.466 |
| MNA | -0.166 | 0.070 | -0.174 | **0.019** |
| Handgrip (kg) | -0.093 | 0.034 | -0.276 | **0.008** |
| BADL | 0.091 | 0.274 | 0.030 | 0.740 |
| IADL | -0.181 | 0.171 | -0.108 | 0.290 |
| HAM-D | 0.016 | 0.030 | 0.032 | 0.598 |
| MoCA | -0.162 | 0.064 | -0.170 | **0.013** |
| Number of drugs | 0.153 | 0.058 | 0.194 | **0.010** |
| L-glutamate (µM) | 0.020 | 0.019 | 0.064 | 0.291 |

**Abbreviations:** BADL, basic activities of daily living (preserved); EFS, Edmonton Frailty Scale; IADL, instrumental activities of daily living (preserved); HAM-D, Hamilton Depression Rating Scale; MNA, Mini Nutritional Assessment; MoCA, Montreal Cognitive Assessment; SE, standard error of β; Std β, standardized β coefficient.

*Suppl. Table H*

|  | **β** | **SE** | **Std β** | **p** |
| --- | --- | --- | --- | --- |
| Constant | 3.299 | 4.184 |  | 0.432 |
| Age (years) | 0.131 | 0.037 | 0.290 | **0.001** |
| Male sex | 0.419 | 0.659 | 0.056 | 0.527 |
| MNA | -0.159 | 0.073 | -0.167 | **0.032** |
| Handgrip (kg) | -0.093 | 0.035 | -0.275 | **0.009** |
| BADL | 0.123 | 0.277 | 0.040 | 0.659 |
| IADL | -0.179 | 0.172 | -0.107 | 0.301 |
| HAM-D | 0.019 | 0.031 | 0.037 | 0.544 |
| MoCA | -0.156 | 0.064 | -0.164 | **0.017** |
| Number of drugs | 0.164 | 0.057 | 0.208 | **0.005** |
| L-glutamine (µM) | 0.001 | 0.003 | 0.017 | 0.786 |

**Abbreviations:** BADL, basic activities of daily living (preserved); EFS, Edmonton Frailty Scale; IADL, instrumental activities of daily living (preserved); HAM-D, Hamilton Depression Rating Scale; MNA, Mini Nutritional Assessment; MoCA, Montreal Cognitive Assessment; SE, standard error of β; Std β, standardized β coefficient.

*Suppl. Table I*

|  | **β** | **SE** | **Std β** | **p** |
| --- | --- | --- | --- | --- |
| Constant | 4.520 | 4.089 |  | 0.272 |
| Age (years) | 0.130 | 0.037 | 0.288 | 0.001 |
| Male sex | 0.504 | 0.663 | 0.068 | 0.449 |
| MNA | -0.173 | 0.071 | -0.181 | 0.016 |
| Handgrip (kg) | -0.093 | 0.035 | -0.277 | 0.008 |
| BADL | 0.082 | 0.279 | 0.027 | 0.770 |
| IADL | -0.174 | 0.171 | -0.104 | 0.311 |
| HAM-D | 0.015 | 0.031 | 0.030 | 0.621 |
| MoCA | -0.157 | 0.064 | -0.165 | 0.016 |
| Number of drugs | 0.159 | 0.058 | 0.202 | 0.007 |
| L-glutamine/L-glutamate | -0.024 | 0.036 | -0.042 | 0.501 |

**Abbreviations:** BADL, basic activities of daily living (preserved); EFS, Edmonton Frailty Scale; IADL, instrumental activities of daily living (preserved); HAM-D, Hamilton Depression Rating Scale; MNA, Mini Nutritional Assessment; MoCA, Montreal Cognitive Assessment; SE, standard error of β; Std β, standardized β coefficient.

**Supplementary Table J.** Correlations between the serum concentration of amino acids and global cognition measures in the elderly cohort stratified in non-frail and frail subgroups according to EFS score. MMSE and MoCA scores were available for 70 non-frail and 48 frail subjects.

|  | **MMSE** | | | | **MoCA** | | | |
| --- | --- | --- | --- | --- | --- | --- | --- | --- |
|  | **Non-frail** | | **Frail** | | **Non-frail** | | **Frail** | |
|  | **r** | **p^a^** | **r** | **p^a^** | **r** | **p^a^** | **r** | **p^a^** |
| **L-aspartate (μM)** | -0.101 | 0.412 | 0.284 | 0.055 | -0.054 | 0.664 | 0.279 | 0.060 |
| **L-asparagine (μM)** | -0.044 | 0.721 | 0.227 | 0.130 | 0.032 | 0.793 | 0.040 | 0.791 |
| **Glycine (μM)** | 0.084 | 0.498 | -0.459 | **0.001** | 0.176 | 0.151 | -0.299 | **0.044** |
| **D-serine (μM)** | -0.161 | 0.191 | -0.193 | 0.198 | -0.125 | 0.311 | 0.009 | 0.951 |
| **L-serine (μM)** | -0.022 | 0.856 | 0.216 | 0.150 | -0.066 | 0.593 | 0.113 | 0.455 |
| **Glycine/L-serine** | 0.115 | 0.351 | -0.501 | **< 0.001** | 0.192 | 0.117 | -0.295 | **0.046** |
| **D-/Total serine (%)** | -0.087 | 0.479 | -0.333 | **0.024** | -0.101 | 0.413 | -0.068 | 0.652 |
| **L-glutamate (μM)** | -0.048 | 0.700 | 0.232 | 0.121 | 0.098 | 0.426 | 0.073 | 0.628 |
| **L-glutamine (μM)** | 0.086 | 0.487 | 0.123 | 0.416 | -0.004 | 0.977 | -0.052 | 0.732 |
| **L-glutamine/L-glutamate** | 0049 | 0.690 | -0.071 | 0.639 | -0.048 | 0.699 | -0.078 | 0.605 |

^a^ Partial correlation adjusted for age and sex.

**Supplementary Table K.** Correlations between the serum concentration of amino acids and the non-cognitive frailty domains in the non-frail group. The number of subjects for which data were available is reported in the second column.

|  | **N** | **L-aspartate** | | **L-asparagine** | | **Glycine** | | **D-serine** | | **L-serine** | | **Glycine/L-serine** | | **D-/Total serine** | | **L-glutamate** | | **L-glutamine** | | **L-glutamine/L-glutamate** | |
| --- | --- | --- | --- | --- | --- | --- | --- | --- | --- | --- | --- | --- | --- | --- | --- | --- | --- | --- | --- | --- | --- |
|  |  | **r** | **p** | **r** | **p** | **r** | **p** | **r** | **p** | **r** | **p** | **r** | **p** | **r** | **p** | **r** | **p** | **r** | **p** | **r** | **p** |
| SF-36 general health | 71 | 0.144 | 0.239 | 0.078 | 0.522 | 0.004 | 0.973 | -0.066 | 0.591 | 0.071 | 0.562 | -0.014 | 0.907 | -0.126 | 0.301 | 0.135 | 0.270 | 0.180 | 0.139 | -0.114 | 0.353 |
| BADL | 72 | 0120 | 0.322 | 0.026 | 0.833 | 0.134 | 0.268 | 0.126 | 0.297 | -0.044 | 0.297 | 0.140 | 0.249 | 0.154 | 0.204 | 0.089 | 0.466 | -0.046 | 0.704 | -0.171 | -0.157 |
| IADL | 72 | 0.158 | 0.191 | 0.041 | 0.736 | -0.197 | 0.102 | 0.020 | 0.868 | 0.062 | 0.608 | -0.205 | 0.089 | -0.066 | 0.585 | 0.100 | 0.411 | 0.037 | 0.762 | -0.077 | 0.525 |
| Number of drugs | 72 | 0.106 | 0.384 | 0.244 | **0.042** | -0.147 | 0.224 | 0.038 | 0.753 | 0.147 | 0.224 | -0.146 | 0.227 | -0.044 | 0.717 | 0.230 | 0.055 | 0.121 | 0.319 | -0.063 | 0.602 |
| MNA | 74 | -0.118 | 0.325 | -0.252 | **0.032** | -0.010 | 0.936 | -0.135 | 0.259 | -0.303 | 0.010 | 0.087 | 0.470 | 0.125 | 0.297 | 0.127 | 0.288 | -0.220 | 0.064 | -0.294 | **0.012** |
| Handgrip (kg) | 74 | 0.061 | 0.610 | -0.069 | 0.565 | 0.117 | 0.329 | -0.011 | 0.928 | -0.021 | 0.858 | 0.075 | 0.530 | -0.043 | 0.718 | 0.113 | 0.344 | 0.125 | 0.295 | -0.082 | 0.494 |
| SPPB total score | 74 | -0.028 | 0.814 | 0.071 | 0.553 | -0.144 | 0.228 | -0.031 | 0.795 | 0.073 | 0.545 | -0.184 | 0.121 | -0.028 | 0.818 | -0.011 | 0.928 | 0.172 | 0.149 | 0.050 | 0.679 |

**Notes:** p-values refer to age- and sex-adjusted partial correlations. **Abbreviations:** BADL, basic activities of daily living (preserved); EFS, Edmonton Frailty Scale; IADL, instrumental activities of daily living (preserved); HAM-D, Hamilton Depression Rating Scale; MMSE, Mini Mental State Examination; MNA, Mini Nutritional Assessment; MoCA, Montreal Cognitive Assessment; SF-36, Short Form Health Survey 36; SPPB, Short Physical Performance Battery.

**Supplementary Table L.** Correlations between the serum concentration of amino acids and the non-cognitive frailty domains in the frail group. The number of subjects for which data were available is reported in the second column.

|  | **N** | **L-aspartate** | | **L-asparagine** | | **Glycine** | | **D-serine** | | **L-serine** | | **Glycine/L-serine** | | **D-/Total serine** | | **L-glutamate** | | **L-glutamine** | | **L-glutamine/L-glutamate** | |
| --- | --- | --- | --- | --- | --- | --- | --- | --- | --- | --- | --- | --- | --- | --- | --- | --- | --- | --- | --- | --- | --- |
|  |  | **r** | **p** | **r** | **p** | **r** | **p** | **r** | **p** | **r** | **p** | **r** | **p** | **r** | **p** | **r** | **p** | **r** | **p** | **r** | **p** |
| SF-36 general health | 46 | -0.059 | 0.702 | 0.252 | 0.099 | -0.098 | 0.528 | -0.073 | 0.636 | 0.194 | 0.207 | -0.156 | 0.311 | -0.183 | 0.235 | -0.124 | 0.421 | 0.199 | 0.196 | 0.280 | 0.065 |
| BADL | 46 | 0.087 | 0.576 | 0.085 | 0.583 | 0.132 | 0.392 | -0.017 | 0.913 | -0.016 | 0.920 | 0.178 | 0.247 | -0.046 | 0.765 | 0.165 | 0.285 | -0.143 | 0.353 | -0.198 | 0.197 |
| IADL | 46 | 0.024 | 0.876 | 0.005 | 0.974 | 0.137 | 0.375 | -0.079 | 0.608 | 0.051 | 0.744 | 0.134 | 0.386 | -0.157 | 0.308 | 0.127 | 0.412 | -0.137 | 0.374 | -0.135 | 0.381 |
| Number of drugs | 49 | 0.052 | 0.728 | 0.077 | 0.606 | 0.202 | 0.173 | -0.095 | 0.524 | 0.014 | 0.928 | 0.225 | 0.128 | -0.022 | 0.882 | 0.070 | 0.638 | 0.016 | 0.916 | -0.075 | 0.617 |
| MNA | 50 | -0.305 | 0.035 | -0.114 | 0.440 | -0.263 | 0.071 | 0.031 | 0.834 | -0.123 | 0.405 | -0.238 | 0.103 | 0.056 | 0.705 | -0.094 | 0.525 | -0.174 | 0.236 | 0.036 | 0.809 |
| Handgrip (kg) | 51 | -0.132 | 0.365 | -0.038 | 0.795 | -0.268 | 0.062 | -0.156 | 0.284 | -0.095 | 0.515 | -0.195 | 0.179 | -0.151 | 0.301 | -0.051 | 0.730 | -0.051 | 0.730 | -0.030 | 0.838 |
| SPPB total score | 51 | 0.029 | 0.845 | 0.219 | 0.130 | -0.087 | 0.552 | -0.100 | 0.492 | 0.247 | 0.088 | -0.222 | 0.126 | -0.268 | 0.062 | -0.105 | 0.473 | 0.209 | 0.150 | 0.201 | 0.166 |

**Notes:** p-values refer to age- and sex-adjusted partial correlations. **Abbreviations:** BADL, basic activities of daily living (preserved); EFS, Edmonton Frailty Scale; IADL, instrumental activities of daily living (preserved); HAM-D, Hamilton Depression Rating Scale; MMSE, Mini Mental State Examination; MNA, Mini Nutritional Assessment; MoCA, Montreal Cognitive Assessment; SF-36, Short Form Health Survey 36; SPPB, Short Physical Performance Battery.

**Supplementary Table M.** Multinomial logistic regression models for frailty phenotype prediction according to Fried criteria adjusted for the effect of age and sex. Non-frail status was set as reference category.

|  | **Outcome** | **β** | **p** |
| --- | --- | --- | --- |
| **L-aspartate (μM)** | Pre-frail | 0.090 | 0.480 |
|  | Frail | 0.124 | 0.427 |
| **L-asparagine (μM)** | Pre-frail | 0.051 | 0.235 |
|  | Frail | 0.045 | 0.396 |
| **Glycine (μM)** | Pre-frail | 0.004 | 0.208 |
|  | Frail | 0.005 | 0.132 |
| **L-serine (μM)** | Pre-frail | 0.005 | 0.709 |
|  | Frail | 0.014 | 0.428 |
| **Glycine/L-serine** | Pre-frail | 0.183 | 0.317 |
|  | Frail | 0.215 | 0.262 |
| **L-glutamate (μM)** | Pre-frail | 0.010 | 0.723 |
|  | Frail | 0.010 | 0.752 |
| **L-glutamine (μM)** | Pre-frail | -0.004 | 0.298 |
|  | Frail | -0.002 | 0.736 |
| **L-glutamate/L-glutamine** | Pre-frail | -0.029 | 0.556 |
|  | Frail | -0.030 | 0.970 |

Symbols: β, regression coefficient.

**Supplementary Table N.** Correlations between the serum concentration of amino acids and age in elderly cohort stratified by sex.

|  | **Females (n = 95 )** | | **Males (n = 30)** | |
| --- | --- | --- | --- | --- |
|  | **Rho** | **p** | **Rho** | **p** |
| **L-aspartate (μM)** | -0.046 | 0.657 | -0.087 | 0.646 |
| **L-asparagine (μM)** | -0.007 | 0.943 | -0.177 | 0.348 |
| **Glycine (μM)** | 0.020 | 0.851 | 0.194 | 0.303 |
| **D-serine (μM)** | 0.315 | **0.002** | 0.416 | **0.022** |
| **L-serine (μM)** | -0.177 | 0.086 | -0.370 | **0.044** |
| **Glycine/L-serine** | 0.127 | **0.220** | 0.463 | **0.010** |
| **D-/Total serine (%)** | 0.430 | **< 0.001** | 0.619 | **< 0.001** |
| **L-glutamate (μM)** | -0.012 | 0.905 | -0.299 | 0.108 |
| **L-glutamine (μM)** | 0.086 | 0.407 | -0.125 | 0.510 |
| **L-glutamine/L-glutamate** | 0.078 | 0.452 | 0.243 | 0.195 |

Abbreviations: Rho, Spearman’s correlation coefficient.

**Supplementary Table O.** Correlations between the serum levels of amino acids and EFS total score in elderly cohort stratified by sex.

|  | **Females (n = 95)** | | **Males (n = 30)** | |
| --- | --- | --- | --- | --- |
|  | **r** | **p**^a^ | **r** | **p**^a^ |
| **L-aspartate (μM)** | -0.011 | 0.919 | 0.283 | 0.171 |
| **L-asparagine (μM)** | -0.039 | 0.717 | -0.023 | 0.911 |
| **Glycine (μM)** | 0.034 | 0.747 | 0.263 | 0.205 |
| **D-serine (μM)** | 0.240 | **0.023** | -0.040 | 0.850 |
| **L-serine (μM)** | -0.009 | 0.936 | 0.275 | 0.183 |
| **Glycine/L-serine** | 0.040 | 0.707 | 0.245 | 0.227 |
| **D-/Total serine (%)** | 0.273 | **0.009** | -0.240 | 0.247 |
| **L-glutamate (μM)** | 0.041 | 0.699 | 0.181 | 0.386 |
| **L-glutamine (μM)** | 0.092 | 0.389 | 0.082 | 0.697 |
| **L-glutamine/L-glutamate** | -0.011 | 0.921 | -0.133 | 0.527 |

^a^ Partial correlation adjusted for age, BMI, diabetes and smoking status.

**Supplementary Table P.** Multiple linear regression parameters for EFS prediction adjusted for the effect of age, nutritional status, handgrip strength, functional independence, depressive symptoms, cognition and number of medications in elderly cohort stratified by sex. Each amino acid was individually added to the model along with clinical predictors. Complete clinical data were available for 84 females and 26 males.

|  | **Females (n = 84)** | | **Males (n = 26)** | |
| --- | --- | --- | --- | --- |
|  | **β** | **p** | **β** | **p** |
| **L-aspartate (μM)** | -0.012 | 0.902 | -0.071 | 0.682 |
| **L-asparagine (μM)** | -0.024 | 0.566 | -0.035 | 0.523 |
| **Glycine (μM)** | -0.003 | 0.140 | -0.003 | 0.315 |
| **D-serine (μM)** | 0.989 | **0.008** | -0.800 | 0.208 |
| **L-serine (μM)** | 0.006 | 0.659 | 0.010 | 0.608 |
| **Glycine/L-serine** | -0.157 | 0.142 | -0.115 | 0.400 |
| **D-/Total serine (%)** | 0.748 | **0.007** | -0.615 | 0.172 |
| **L-glutamate (μM)** | 0.025 | 0.265 | 0.005 | 0.906 |
| **L-glutamine (μM)** | 0.001 | 0.805 | 0.001 | 0.894 |
| **L-glutamate/L-glutamine** | -0.031 | 0.467 | -0.036 | 0.637 |

**Supplementary Table Q.** Correlations between the serum concentration of D-Ser, D-/Total Ser and frailty domains according to EFS in the elderly cohort stratified by sex. The number of subjects for which data were available is reported in the first column (F, females; M, males).

|  | **Females** | | | | **Males** | | | |
| --- | --- | --- | --- | --- | --- | --- | --- | --- |
|  | **D-serine** | | **D-/Total serine** | | **D-serine** | | **D-/Total serine** | |
|  | **r** | **p^a^** | **r** | **p^a^** | **r** | **p^a^** | **r** | **p^a^** |
| MMSE (91 F, 27 M) | -0.222 | **0.035** | -0.289 | **0.006** | -0.289 | 0.153 | 0.170 | 0.406 |
| MoCA (91 F, 27 M) | -0.128 | 0.228 | -0.235 | **0.025** | -0.034 | 0.867 | 0.326 | 0.105 |
| SF-36 general health (90 F, 27 M) | -0.088 | 0.413 | -0.244 | **0.021** | -0.108 | 0.600 | 0.161 | 0.433 |
| BADL (90 F, 28 M) | 0.029 | 0.791 | 0.020 | 0.856 | 0.007 | 0.972 | -0.186 | 0.353 |
| IADL (90 F, 28 M) | -0.036 | 0.741 | -0.098 | 0.361 | -0.131 | 0.514 | -0.274 | 0.166 |
| Number of drugs (93 F, 28 M) | 0.062 | 0.557 | 0.111 | 0.291 | -0.083 | 0.680 | -0.273 | 0.169 |
| MNA (94 F, 30 M) | -0.047 | 0.654 | -0.003 | 0.980 | -0.224 | 0.243 | 0.121 | 0.533 |
| HAM-D (90 F, 28 M) | -0.037 | 0.730 | 0.140 | 0.191 | 0.018 | 0.928 | -0.124 | 0.539 |
| Handgrip (kg) (95 F, 30 M) | -0.037 | 0.726 | -0.110 | 0.293 | -0.280 | 0.141 | -0.021 | 0.914 |
| SPPB total score (95 F, 30 M) | -0.108 | 0.299 | -0.209 | **0.043** | -0.072 | 0.709 | -0.218 | 0.256 |

^a^ Age-adjusted partial correlation

**Abbreviations:** BADL, basic activities of daily living (preserved); EFS, Edmonton Frailty Scale; IADL, instrumental activities of daily living (preserved); HAM-D, Hamilton Depression Rating Scale; MMSE, Mini Mental State Examination; MNA, Mini Nutritional Assessment; MoCA, Montreal Cognitive Assessment; SF-36, Short Form Health Survey 36; SPPB, Short Physical Performance Battery.

**Supplementary Table R.** Correlations between the serum L-aspartate concentration and frailty domains according to EFS in the elderly cohort stratified by sex. The number of subjects for which data were available is reported in the first column (F, females; M, males).

|  | **Females** | | **Males** | |
| --- | --- | --- | --- | --- |
|  | **r** | **p^a^** | **r** | **p^a^** |
| MMSE (91 F, 27 M) | 0.108 | 0.309 | -0.123 | 0.548 |
| MoCA (91 F, 27 M) | 0.132 | 0.217 | -0.123 | 0.548 |
| SF-36 general health (90 F, 27 M) | 0.029 | 0.787 | 0.042 | 0.837 |
| BADL (90 F, 28 M) | 0.114 | 0.289 | -0.231 | 0.247 |
| IADL (90 F, 28 M) | 0.054 | 0.613 | 0.006 | 0.976 |
| Number of drugs (93 F, 28 M) | 0.087 | 0.409 | 0.238 | 0.232 |
| MNA (94 F, 30 M) | 0.169 | 0.106 | -0.478 | **0.009** |
| HAM-D (90 F, 28 M) | 0.005 | 0.965 | -0.104 | 0.607 |
| Handgrip (kg) (95 F, 30 M) | 0.012 | 0.909 | -0.253 | 0.185 |
| SPPB total score (95 F, 30 M) | -0.006 | 0.951 | -0.295 | 0.120 |

^a^ age-adjusted partial correlation

**Abbreviations:** BADL, basic activities of daily living (preserved); EFS, Edmonton Frailty Scale; IADL, instrumental activities of daily living (preserved); HAM-D, Hamilton Depression Rating Scale; MMSE, Mini Mental State Examination; MNA, Mini Nutritional Assessment; MoCA, Montreal Cognitive Assessment; SF-36, Short Form Health Survey 36; SPPB, Short Physical Performance Battery.

**Supplementary Table S**. Correlations between the serum L-asparagine concentration and frailty domains according to EFS in the elderly cohort stratified by sex. The number of subjects for which data were available is reported in the first column (F, females; M, males).

|  | **Females** | | **Males** | |
| --- | --- | --- | --- | --- |
|  | **r** | **p^a^** | **r** | **p^a^** |
| MMSE (91 F, 27 M) | 0.147 | 0.167 | -0.174 | 0.395 |
| MoCA (91 F, 27 M) | 0.019 | 0.861 | 0.043 | 0.834 |
| SF-36 general health (90 F, 27 M) | 0.180 | 0.092 | 0.029 | 0.890 |
| BADL (90 F, 28 M) | 0.077 | 0.472 | -0.098 | 0.628 |
| IADL (90 F, 28 M) | 0.091 | 0.397 | -0.256 | 0.198 |
| Number of drugs (93 F, 28 M) | 0.061 | 0.561 | 0.350 | 0.073 |
| MNA (94 F, 30 M) | -0.152 | 0.145 | -0.098 | 0.613 |
| HAM-D (90 F, 28 M) | -0.338 | **0.001** | 0.225 | 0.258 |
| Handgrip (kg) (95 F, 30 M) | -0.030 | 0.711 | -0.110 | 0.570 |
| SPPB total score (95 F, 30 M) | 0.178 | 0.086 | 0.007 | 0.971 |

^a^ age-adjusted partial correlation

**Abbreviations:** BADL, basic activities of daily living (preserved); EFS, Edmonton Frailty Scale; IADL, instrumental activities of daily living (preserved); HAM-D, Hamilton Depression Rating Scale; MMSE, Mini Mental State Examination; MNA, Mini Nutritional Assessment; MoCA, Montreal Cognitive Assessment; SF-36, Short Form Health Survey 36; SPPB, Short Physical Performance Battery.

**Supplementary Table T.** Correlations between the serum glycine concentration and frailty domains according to EFS in the elderly cohort stratified by sex. The number of subjects for which data were available is reported in the first column (F, females; M, males).

|  | **Females** | | **Males** | |
| --- | --- | --- | --- | --- |
|  | **r** | **p^a^** | **r** | **p^a^** |
| MMSE (91 F, 27 M) | -0.205 | 0.053 | 0.209 | 0.305 |
| MoCA (91 F, 27 M) | -0.149 | 0.160 | 0.171 | 0.403 |
| SF-36 general health (90 F, 27 M) | -0.036 | 0.741 | -0.104 | 0.612 |
| BADL (90 F, 28 M) | 0.120 | 0.263 | 0.215 | 0.282 |
| IADL (90 F, 28 M) | 0.069 | 0.524 | -0.372 | 0.056 |
| Number of drugs (93 F, 28 M) | 0.104 | 0.323 | -0.133 | 0.508 |
| MNA (94 F, 30 M) | -0.093 | 0.377 | -0.380 | **0.042** |
| HAM-D (90 F, 28 M) | 0.216 | **0.042** | 0.013 | 0.947 |
| Handgrip (kg) (95 F, 30 M) | -0-072 | 0.489 | -0.003 | 0.988 |
| SPPB total score (95 F, 30 M) | -0.090 | 0.391 | -0.234 | 0.222 |

^a^ age-adjusted partial correlation

**Abbreviations:** BADL, basic activities of daily living (preserved); EFS, Edmonton Frailty Scale; IADL, instrumental activities of daily living (preserved); HAM-D, Hamilton Depression Rating Scale; MMSE, Mini Mental State Examination; MNA, Mini Nutritional Assessment; MoCA, Montreal Cognitive Assessment; SF-36, Short Form Health Survey 36; SPPB, Short Physical Performance Battery.

**Supplementary Table U.** Correlations between the serum L-serine concentration and frailty domains according to EFS in the elderly cohort stratified by sex. The number of subjects for which data were available is reported in the first column (F, females; M, males).

|  | **Females** | | **Males** | |
| --- | --- | --- | --- | --- |
|  | **r** | **p^a^** | **r** | **p^a^** |
| MMSE (91 F, 27 M) | 0.150 | 0.158 | -0.170 | 0.406 |
| MoCA (91 F, 27 M) | 0.093 | 0.383 | -0.243 | 0.231 |
| SF-36 general health (90 F, 27 M) | 0.204 | 0.055 | -0.142 | 0.489 |
| BADL (90 F, 28 M) | -0.045 | 0.674 | 0.082 | 0.685 |
| IADL (90 F, 28 M) | 0.018 | 0.870 | 0.073 | 0.717 |
| Number of drugs (93 F, 28 M) | -0.004 | 0.968 | 0.295 | 0.135 |
| MNA (94 F, 30 M) | -0.114 | 0.276 | -0.383 | **0.040** |
| HAM-D (90 F, 28 M) | -0.226 | 0.033 | 0.152 | 0.448 |
| Handgrip (kg) (95 F, 30 M) | 0.106 | 0.311 | -0.265 | 0.165 |
| SPPB total score (95 F, 30 M) | 0.150 | 0.148 | 0.018 | 0.927 |

^a^ age-adjusted partial correlation

**Abbreviations:** BADL, basic activities of daily living (preserved); EFS, Edmonton Frailty Scale; IADL, instrumental activities of daily living (preserved); HAM-D, Hamilton Depression Rating Scale; MMSE, Mini Mental State Examination; MNA, Mini Nutritional Assessment; MoCA, Montreal Cognitive Assessment; SF-36, Short Form Health Survey 36; SPPB, Short Physical Performance Battery.

**Supplementary Table V.** Correlations between the serum glycine/L-serine ratio and frailty domains according to EFS in the elderly cohort stratified by sex. The number of subjects for which data were available is reported in the first column (F, females; M, males).

|  | **Females** | | **Males** | |
| --- | --- | --- | --- | --- |
|  | **r** | **p^a^** | **r** | **p^a^** |
| MMSE (91 F, 27 M) | -0.299 | **0.004** | 0.315 | 0.117 |
| MoCA (91 F, 27 M) | -0.149 | 0.160 | 0.289 | 0.152 |
| SF-36 general health (90 F, 27 M) | -0.098 | 0.363 | -0.010 | 0.960 |
| BADL (90 F, 28 M) | 0.159 | 0.135 | 0.167 | 0.405 |
| IADL (90 F, 28 M) | 0.081 | 0.448 | -0.422 | **0.028** |
| Number of drugs (93 F, 28 M) | 0.132 | 0.209 | -0.194 | 0.332 |
| MNA (94 F, 30 M) | -0.028 | 0.793 | -0.358 | 0.056 |
| HAM-D (90 F, 28 M) | 0.334 | **0.001** | -0.008 | 0.967 |
| Handgrip (kg) (95 F, 30 M) | -0.097 | 0.354 | 0.051 | 0.793 |
| SPPB total score (95 F, 30 M) | -0.148 | 0.153 | -0.330 | 0.080 |

^a^ age-adjusted partial correlation

**Abbreviations:** BADL, basic activities of daily living (preserved); EFS, Edmonton Frailty Scale; IADL, instrumental activities of daily living (preserved); HAM-D, Hamilton Depression Rating Scale; MMSE, Mini Mental State Examination; MNA, Mini Nutritional Assessment; MoCA, Montreal Cognitive Assessment; SF-36, Short Form Health Survey 36; SPPB, Short Physical Performance Battery.

**Supplementary Table W.** Correlations between the serum L-glutamate concentration and frailty domains according to EFS in the elderly cohort stratified by sex. The number of subjects for which data were available is reported in the first column (F, females; M, males).

|  | **Females** | | **Males** | |
| --- | --- | --- | --- | --- |
|  | **r** | **p^a^** | **r** | **p^a^** |
| MMSE (91 F, 27 M) | 0.112 | 0.292 | -0.106 | 0.605 |
| MoCA (91 F, 27 M) | 0.085 | 0.424 | -0.073 | 0.721 |
| SF-36 general health (90 F, 27 M) | 0.007 | 0.951 | -0.043 | 0.836 |
| BADL (90 F, 28 M) | 0.115 | 0.281 | 0.062 | 0.757 |
| IADL (90 F, 28 M) | 0.061 | 0.570 | 0.042 | 0.834 |
| Number of drugs (93 F, 28 M) | 0.150 | 0.154 | 0.393 | **0.043** |
| MNA (94 F, 30 M) | 0.025 | 0.812 | -0.356 | 0.058 |
| HAM-D (90 F, 28 M) | 0.081 | 0.452 | 0.092 | 0.647 |
| Handgrip (kg) (95 F, 30 M) | 0.010 | 0.922 | -0.061 | 0.755 |
| SPPB total score (95 F, 30 M) | -0.119 | 0.254 | -0.167 | 0.387 |

^a^ age-adjusted partial correlation

**Abbreviations:** BADL, basic activities of daily living (preserved); EFS, Edmonton Frailty Scale; IADL, instrumental activities of daily living (preserved); HAM-D, Hamilton Depression Rating Scale; MMSE, Mini Mental State Examination; MNA, Mini Nutritional Assessment; MoCA, Montreal Cognitive Assessment; SF-36, Short Form Health Survey 36; SPPB, Short Physical Performance Battery.

**Supplementary Table X.** Correlations between the serum L-glutamine concentration and frailty domains according to EFS in the elderly cohort stratified by sex. The number of subjects for which data were available is reported in the first column (F, females; M, males).

|  | **Females** | | **Males** | |
| --- | --- | --- | --- | --- |
|  | **r** | **p^a^** | **r** | **p^a^** |
| MMSE (91 F, 27 M) | 0.172 | 0.105 | -0.099 | 0.630 |
| MoCA (91 F, 27 M) | -0.062 | 0.560 | 0.042 | 0.839 |
| SF-36 general health (90 F, 27 M) | 0.176 | 0.099 | 0.080 | 0.697 |
| BADL (90 F, 28 M) | -0.147 | 0.169 | 0.111 | 0.582 |
| IADL (90 F, 28 M) | -0.101 | 0.347 | -0.033 | 0.868 |
| Number of drugs (93 F, 28 M) | 0.048 | 0.651 | 0.212 | 0.289 |
| MNA (94 F, 30 M) | -0.259 | 0.012 | -0.023 | 0.905 |
| HAM-D (90 F, 28 M) | -0.227 | **0.032** | 0.159 | 0.429 |
| Handgrip (kg) (95 F, 30 M) | 0.044 | 0.674 | -0.016 | 0.933 |
| SPPB total score (95 F, 30 M) | 0.103 | 0.325 | 0.218 | 0.255 |

^a^ age-adjusted partial correlation

**Abbreviations:** BADL, basic activities of daily living (preserved); EFS, Edmonton Frailty Scale; IADL, instrumental activities of daily living (preserved); HAM-D, Hamilton Depression Rating Scale; MMSE, Mini Mental State Examination; MNA, Mini Nutritional Assessment; MoCA, Montreal Cognitive Assessment; SF-36, Short Form Health Survey 36; SPPB, Short Physical Performance Battery.

**Supplementary Table Y.** Correlations between the serum L-glutamine/L-glutamate concentration and frailty domains according to EFS in the elderly cohort stratified by sex. The number of subjects for which data were available is reported in the first column (F, females; M, males).

|  | **Females** | | **Males** | |
| --- | --- | --- | --- | --- |
|  | **r** | **p^a^** | **r** | **p^a^** |
| MMSE (91 F, 27 M) | -0.009 | 0.936 | 0.040 | 0.848 |
| MoCA (91 F, 27 M) | -0.094 | 0.376 | 0.121 | 0.557 |
| SF-36 general health (90 F, 27 M) | 0.043 | 0.692 | 0.164 | 0.423 |
| BADL (90 F, 28 M) | -0.192 | 0.071 | -0.058 | 0.775 |
| IADL (90 F, 28 M) | -0.083 | 0.438 | -0.079 | 0.696 |
| Number of drugs (93 F, 28 M) | -0.098 | 0.355 | -0.187 | 0.350 |
| MNA (94 F, 30 M) | -0.143 | 0.172 | 0.301 | 0.113 |
| HAM-D (90 F, 28 M) | -0.178 | 0.095 | -0.048 | 0.810 |
| Handgrip (kg) (95 F, 30 M) | -0.059 | 0.574 | 0.092 | 0.634 |
| SPPB total score (95 F, 30 M) | 0.175 | 0.091 | 0.165 | 0.393 |

^a^ age-adjusted partial correlation

**Abbreviations:** BADL, basic activities of daily living (preserved); EFS, Edmonton Frailty Scale; IADL, instrumental activities of daily living (preserved); HAM-D, Hamilton Depression Rating Scale; MMSE, Mini Mental State Examination; MNA, Mini Nutritional Assessment; MoCA, Montreal Cognitive Assessment; SF-36, Short Form Health Survey 36; SPPB, Short Physical Performance Battery.
